# Graphene Oxide Membranes for Recovery of Catalytically Depolymerized Lignin Monomers and Oligomers

**DOI:** 10.1002/cssc.71123

**Published:** 2026-10-20

**Authors:** Natechanok Yutthasaksunthorn, Dhirendra Kumar Mishra, Yinglei Han, Muskan Sonker, Scott A. Sinquefield, Blake A. Simmons, Hemant Choudhary, Sankar Nair

**Affiliations:** ^1^ School of Chemical & Biomolecular Engineering Georgia Institute of Technology Atlanta Georgia USA; ^2^ Deconstruction Division Joint BioEnergy Institute Emeryville California USA; ^3^ Department of Bioresource and Environmental Security Sandia National Laboratories Livermore California USA; ^4^ Renewable Bioproducts Institute Georgia Institute of Technology Atlanta Georgia USA; ^5^ Biological Systems and Engineering Division Lawrence Berkeley National Laboratory Berkeley California USA

**Keywords:** biomass, catalytic lignin, depolymerization, graphene oxide, membrane separation, metal‐free carbon catalyst

## Abstract

Efficient lignin valorization requires not only catalytic depolymerization but also a scalable, aqueous separation route that isolates bioavailable low‐molecular‐weight (LMW) products from recalcitrant oligomers/polymers. Here, oxidative depolymerization of poplar lignin was coupled with cross‐flow nanofiltration using reduced‐graphene‐oxide (rGO) membranes (molecular‐weight cut‐off or MWCO ≈ 470 Da). Across six depolymerization liquors spanning pH 2.5–9.2 and distinct catalytic histories (of polyoxometalate (POM), NaOH, graphene oxide, and control), rGO membranes consistently rejected ≥90% of the >1.6 kDa fraction, while feed pH governed LMW partitioning. Acidic liquors favored a steric‐sieving regime that enriched aliphatic and aromatic monomers in the permeate (separation factors ≈5–6 relative to the high‐MW fraction), whereas alkaline conditions induced electrostatic exclusion, co‐retaining anionic acids with oligomers and suppressing monomer enrichment. Mechanistic studies with model mixtures showed that dilution sharpened selectivity by mitigating concentration polarization and ionic‐strength screening and that aromatics can modulate charge‐based exclusion through competitive interactions within graphitic nanochannels. Continuous diafiltration of the monomer‐rich liquor for 250 h sustained stable flux (~11 ± 1 L m^−2^ h^−1^) and selectivity, enabling complete recovery of aliphatic and aromatic monomers and LMW fractions while concentrating high‐MW fractions. These results establish rGO membranes as a robust, water‐based separation platform for MW‐resolved lignin fractionation.

## Graphical abstract

Poplar lignin is oxidatively depolymerized to produce water‐soluble liquor containing monomeric acids and low‐molecular‐weight (LMW) oligomers. When the depolymerization product liquor fractionated through an ultrathin reduced‐graphene‐oxide laminate, the 2‐D nano‐channels transmitted the valuable ≤1.6 kDa species into the permeate, while high‐MW fragments are retained for further valorization—demonstrating an all‐aqueous, catalyst‐agnostic route to lignin monomer isolation.

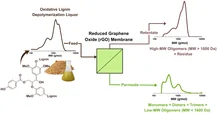

## 1 Introduction

Lignin—the largest renewable source of aromatic carbon—accounts for nearly 30% of biomass carbon yet remains under‐exploited due to its structural heterogeneity and chemical resist‍‍‍ance to depolymerization [1, 2]. Catalytic depolymerization methods, including oxidative, reductive, and base‐catalyzed approaches, have advanced substantially in breaking down lignin into low‐molecular‐weight (LMW) products [3, 4, 5, 6, 7] such as phenolics, aldehydes, and carboxylic acids. Prior studies have established alkaline aerobic oxidation as an effective route for converting technical lignins and lignin‐containing pulping liquors into value‐added low‐molecular‐weight products. Early kinetic studies [8] demonstrated that vanillin formation and subsequent degradation during O_2_ oxidation of kraft lignin are strongly governed by reaction conditions. Building on this foundation, oxidative lignin valorization was extended to industrial *Eucalyptus globulus* kraft and sulfite liquors, demonstrating the production of vanillin and syringaldehyde [9] from technically relevant feedstocks. Despite these successes, the resulting product streams invariably contain a broad MW distribution [10, 11, 12]: reactive monomers, dimers, oligomers, and recalcitrant polymers. This polydispersity hinders downstream valorization due to many reasons, notably the repolymerization or condensation of unstable intermediates, thus lowering monomer/oligomer yields. Subsequent work broadened the product scope beyond aromatic aldehydes to include other phenolic monomers and LMV carboxylic acids while also developing downstream recovery strategies based on adsorption, chromatography, and membrane‐assisted separations [13, 14]. Collectively, these studies underscore that oxidative depolymerization and product separation are intrinsically coupled challenges and that efficient fractionation [15, 16] of chemically and molecularly diverse lignin‐derived streams is essential for effective lignin valorization.

The separation of depolymerized lignin products by MW has therefore emerged as a critical bottleneck in lignin valorization. Early strategies relied on solvent extraction, distillation, and chromatographic fractionation [17, 18, 19, 20], which provided valuable insights but were limited by high solvent demand, poor scalability, and narrow operating windows. Membrane‐based separations introduced in the last decade addressed some of these challenges, offering energy‐efficient size cut‐offs in aqueous and organic media [21, 22, 23, 24]. However, most commercial nanofiltration (NF) membranes suffer from fouling and chemical instability when exposed to real lignin‐containing liquors or do not have sufficient resolution to separate monomers from oligomers [25, 26, 27]. Recent work has demonstrated MW‐based lignin recovery/fractionation from black liquor and from organosolv lignin streams using polymeric or ceramic membranes. However, systematic monomer/oligomer‐resolved MW fractionation of aqueous oxidative depolymerization liquors, where selectivity is strongly governed by pH‐dependent ionization, remains limited [26, 27, 28, 29, 30]. Such MW‐targeted fractionation is especially important for integration with bioconversion [11]. Microbial pathways for upgrading lignin products typically require relatively uniform LMW streams; prolonged coexistence with higher‐MW oligomers promotes instability and inhibits microbial assimilation [31, 32]. Delivering well‐defined molecular‐weight fractions, with monomer‐rich permeates and oligomer/polymer retentates, would not only stabilize intermediates but also align product streams with distinct chemical and biological upgrading routes, thereby accelerating the development of integrated lignin biorefineries.

Graphene oxide (GO) and reduced graphene oxide (rGO) membranes provide a strongly emerging route to address the above challenges. Their stacked laminates (nanosheets) create two‐dimensional sub‐nanometer channels with tunable spacing, exceptional chemical/thermal stability, and favorable interactions with aromatic solutes [28, 33, 34, 35, 36, 37, 38, 39]. Several recent works from rGO membranes fractionate lignin‐rich kraft black liquor, rejecting oligomers under highly caustic (pH ≈ 13) and saline conditions for hundreds of hours without loss of performance [28, 37]. These attributes make rGO membranes uniquely suited to fractionating real oxidative depolymerization liquors into MW‐defined product classes, enabling both monomer recovery and retentate valorization. Figure 1 shows the concept, illustrated by the oxidative catalytic depolymerization of poplar lignin followed by continuous cross‐flow nanofiltration over an rGO membrane that separates the water‐soluble product liquor into a monomer/low‐MW permeate (< 1600 Da) and a high‐MW retentate for recycle or other uses. In this work, we address the aforementioned literature gap by coupling the oxidative depolymerization of poplar lignin with systematic water‐based nanofiltration using few‐layer rGO membranes. We first investigate the fractionation of six distinct catalytic liquors across a wide pH range (2.5–9.2) to elucidate the transition between steric sieving and electrostatic exclusion regimes. We then employ model feed solutions to isolate the effects of the solute concentration and aromaticity on membrane selectivity. Finally, we demonstrate the robustness of the rGO platform through a 250 h continuous diafiltration study, establishing an energy‐efficient, single‐step route for isolating bioavailable monomers while preserving high‐MW residues for material valorization.

**FIGURE 1:**
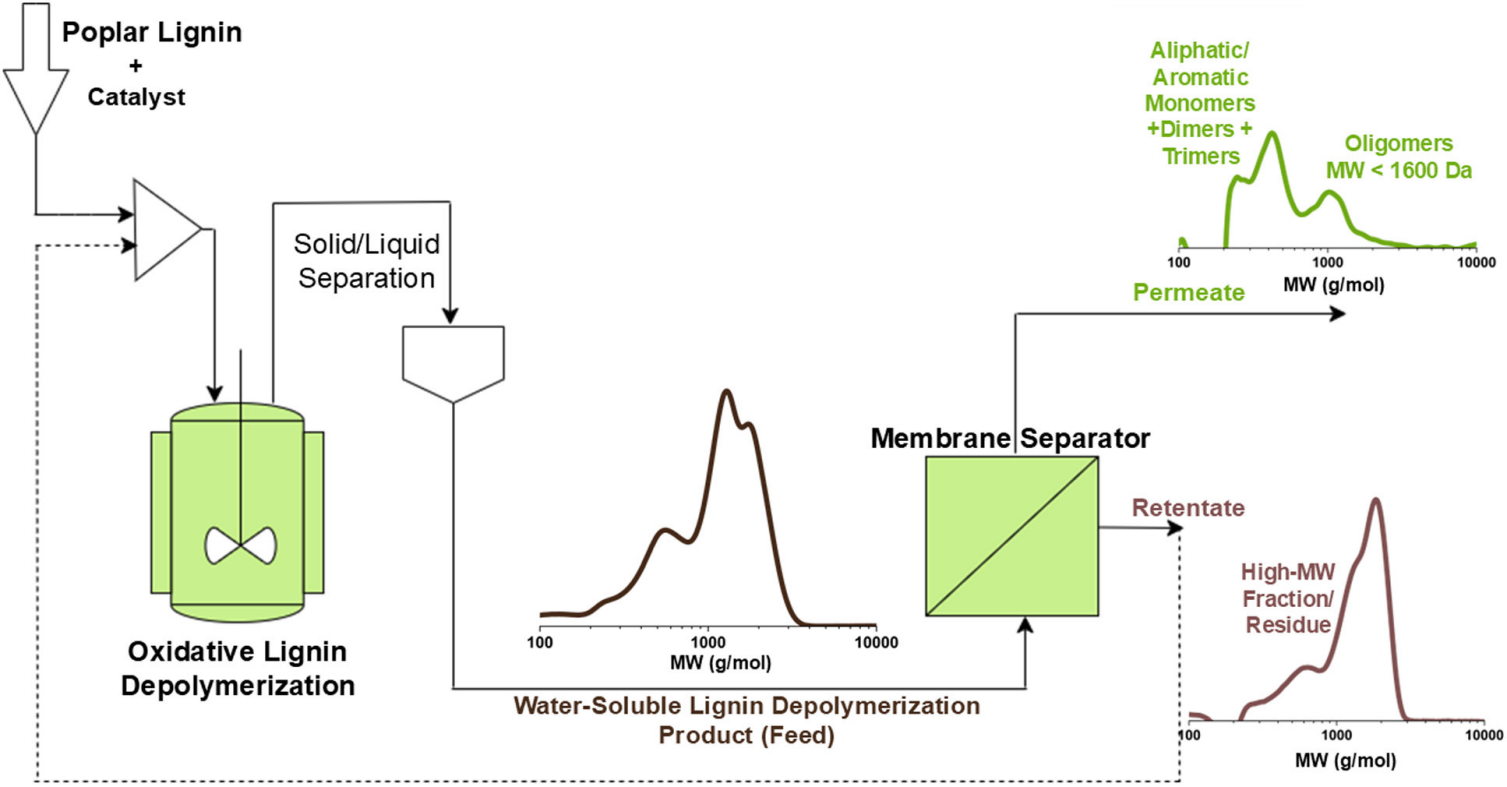
Schematic of the proposed concept for lignin valorization combining catalytic depolymerization and membrane‐based recovery of low‐molecular‐weight (LMW) lignin monomers and oligomers.

## 2 Experimental Section

### 2.1 Materials

Poplar lignin was extracted using cholinium lysinate (ChLys) ionic liquid pretreatment, following the reported procedure [40]. Poplar lignin (unpretreated) and catalytic polyoxometalates (POM) were prepared as described previously [40, 41]. Graphite powder (synthetic, ≤45 µm), concentrated H_2_SO4 (98 %), KMnO_4_, K_2_S_2_O_8_, P_2_O_5_, HCl, and NaOH were purchased from Sigma‐Aldrich and used as received. De‐ionized water (conductivity 4.5 µS cm^−1^, 25 °C) was produced in‐house (Thermo Scientific RO 7128). Ultrafiltration supports, PVDF (polyvinylidene fluoride) and PES (polyethersulfone) substrates (47 mm, 0.2 µm pore size) were purchased from Sterlitech Corp. Poly(styrene sulfonate) sodium salt standards (246–30 000 Da) used for GPC calibration were purchased from Agilent. Authentic standards of aliphatic acids and aromatic monomers (0.01–1.0 g L^−1^) were obtained from Sigma‐Aldrich and used for HPLC quantification. All solvents and mobile‐phase additives for chromatography, including acetonitrile, ammonium acetate, and formic acid, were HPLC grade.

### 2.2 Preparation of Polyoxometalate (POM) Catalyst

The POM catalyst was synthesized as reported by us previously [41]. Phosphotungstic acid (1 equiv.) was dissolved in water. The solution was cooled with an ice bath and vigorously stirred. An aqueous solution of cholinium hydroxide (3 equiv.) was added dropwise to the mixture, forming a white precipitate. The mixture was allowed to stir for 2 h before the precipitate was collected by vacuum filtration. The solid was washed three times with water before being dried in a furnace at 40 °C overnight.

### 2.3 Preparation of Graphene Oxide (GO) and Reduced Graphene Oxide (rGO) Suspensions

GO synthesis was performed. Graphene oxide was produced by a modified Hummers protocol that we have detailed previously [28]. After oxidation, the slurry was centrifuged (4000 rpm, 15 min), and the supernatant was discarded. The sediment was washed sequentially with deionized (DI) water, 1 M HCl, and finally DI water until the supernatant reached a near‐neutral pH, ensuring complete removal of Mn^2+^ species. Exfoliation was achieved by sonication of the washed solids in DI water for 2 h. The dispersion was centrifuged as above; the red–brown supernatant (well‐exfoliated GO) was collected, whereas the residue was re‐dispersed, sonicated, and centrifuged repetitively until the supernatant became pale yellow. GO concentration was determined gravimetrically, and the stock suspension was diluted to 1.0 g L^−1^ and stored in the dark at 4 °C for rGO synthesis. To obtain partially reduced GO, 0.25 g of NaOH was added to 100 mL of the 1.0 g L^−1^ GO suspension. The mixture was stirred vigorously for 30 min and then refluxed at 100 °C for 2 h. After cooling, a 30‐min bath sonication produced a stable black suspension (≈1 g L^−1^ rGO).

### 2.4 Fabrication of GO and rGO Nanofiltration Membranes

GO or rGO suspensions (1 mg/mL) were diluted to 500 mL with DI water, stirred for 1 min, and ultrasonicated for 5 min. The dispersion was vacuum‐filtered through the pre‐wetted porous poly(vinylidene fluoride) (PVDF) or polyethersulfone (PES) substrate coupons held in a Büchner funnel. After filtration, the laminate was rinsed once with DI water to remove loosely bound flakes and dried in air overnight. Membranes fabricated from pristine GO are designated GO‐PES or GO‐PVDF; those from NaOH‐reduced GO are labeled rGO‐PES or rGO‐PVDF. For prior performance experiment, membranes were compacted in DI water at 30 bar for rGO/PVDF and 50 bar for rGO/PES (crossflow cell) until the water flux stabilized (< 5 % change/h; typically 24–48 h). Compact films are denoted as rGO.

### 2.5 Estimation of Membrane Molecular‐Weight Cut‐Off (MWCO)

The molecular‐weight cut‐off (MWCO) of each membrane was estimated in the standard manner, that is, measuring the rejections of test molecules with a range of MWs and identifying the MW at which the membrane exhibits a 90% rejection rate. For aqueous measurements, the experiments were conducted with a 0.3 mM dye concentration at 25 °C and a transmembrane pressure differential of 30 bar. The selected dyes for this analysis included Methyl Blue (800 Da, acidic), Congo Red (700 Da, basic), Allura Red AC (500 Da, acidic), and Cresol Red (400 Da, basic). A 0.3 mM dye solution was filtered at 25 °C and 30 bar; permeate and feed concentrations were quantified by UV–Vis spectroscopy (Agilent 8453). The solute showing 90 % rejection defined the MWCO. Basic dyes were removed from the membrane by soaking in 1 N HCl for 30 min, whereas acidic dyes were stripped with 1 N NaOH under identical conditions; the membrane was subsequently rinsed with DI water for at least 12 h under cross‐flow to restore its baseline state. In addition to reporting a single MWCO value (Figure S1), we estimated an effective pore size distribution using the group’s established microstructure‐permeation framework for rGO membranes. Specifically, water permeance and neutral‐solute (or dye‐based) rejection trends were interpreted using a slit‐pore transport model to extract statistically distributed microstructural parameters (e.g., effective pore radius distributions) rather than a single deterministic pore size. This distribution‐based description is appropriate for laminated 2D membranes, where transport occurs through an ensemble of pathways that vary due to nanosheet stacking heterogeneity, edge gaps, and compaction history [42, 43].

### 2.6 Lignin Depolymerization

All reactions were carried out in a 50 mL, T‐316 SS Parr 4593 bench‐top autoclave equipped with a PTFE liner, dual‐turbine impeller (0–600 rpm), J‐type thermocouple, and a 3000 psi pressure rating. Prior to each run, the vessel was leak‐tested with N_2_ at 20 bar, rinsed with deionized (DI) water, and dried at 105 °C overnight. Poplar lignin (0.35 g, dry basis) was slurried in 50 mL of DI water together with the desired catalyst: NaOH (0.10 g), polyoxometalate (POM, 0.10 g), and graphene oxide (GO, 0.10 g) (Figure S6). Where specified, 1 wt% H_2_O_2_ (relative to lignin) was added as an oxidative co‐reagent. The suspension was stirred for 10 min to ensure homogeneous dispersion and then transferred to the reactor. The vessel was sealed and purged three times with the target head‐space gas (O_2_ or N_2_) by pressurizing to the target pressure bar and venting to atmospheric pressure and finally pressurized to the target pressure with the same gas. Under continuous stirring (500 rpm), the temperature was ramped from ambient to the set point (140 or 160 °C) at 5 °C/min. The reaction was then held isothermally for 1 h. Head‐space pressure was maintained at 10 bar throughout. At the end of the reaction, the heater was switched off, and the vessel was immersed in an ice bath to reach <30 °C within 5 min. Gas was vented slowly, the slurry was filtered (0.45 µm PTFE), and both filtrate and solid residue were stored at 4 °C for analysis.

### 2.7 Nanofiltration and Diafiltration

Continuous nanofiltration and diafiltration measurements were performed in a Sterlitech CF047 stainless‐steel crossflow cell (half diameter of the effective area, 2.35 cm, channel height ≈ 0.19 cm). The module was mounted in a closed loop driven by a magnetically coupled gear pump set to 190 mL min^−1^ (3.17 cm^3^/s), which—together with the channel geometry supplied by the manufacturer—corresponds to a cross‐flow velocity of 7.06 cm/s and Reynolds numbers of ≈240 for the water as solvent (laminar regime). The feed and retentate lines were fitted with a 0–60 bar stainless‐steel pressure transducer. All wetted parts (piping, fittings, cell body, and gaskets) were 316 SS or PTFE to eliminate oxidative corrosion during lignin depolymerization product runs. For diafiltration, DI water was metered into the feed reservoir at a rate equal to the permeate outflow, keeping the transmembrane pressure constant (30 bar) and enabling steady‐state assessment of membrane selectivity under progressively decreasing monomer and oligomer solute concentration. Surface and cross‐sectional morphologies were examined by FE‐SEM (Hitachi SU8230, Au:Pd 60:40 sputter coat, Quorum Q‐150 VES Plus), while surface chemistry was analyzed by XPS (Thermo K‐Alpha, monochromatic Al Kα source, detection depth ~5 nm). SEM assessed structural integrity (surface roughness and delamination), and XPS quantified elemental composition before and after use in the separation measurements.

### 2.8 Gel‐Permeation Chromatography (GPC)

Molecular‐weight distributions were measured on an Agilent 1260 Infinity II equipped with a PL‐Aquagel OH mixed‐M column (300 mm × 7.5 mm, 8 µm). The eluent was 0.20 wt% sodium azide in water, delivered at 1.0 mL min^−1^ and held at 40 °C. Lignin‐derived liquor was filtered through a 0.20 µm PTFE syringe filter and injected. Elution was monitored with a UV detector at 280 nm. Apparent weight‐average (MW) molar masses were obtained by calibration with polystyrene sulfonate sodium salt standards (246–30 000 Da). Gaussian fitting of the chromatograms provided the area fractions used to calculate separation factors for (i) monomer + dimer + trimer (< 1 kDa), (ii) oligomer (1‍–‍1.6 kDa), and (iii) high MW (> 1.6 kDa) fractions. The GPC calibration was carried out as follows: Absolute molecular weights were assigned from a nine‐point calibration constructed with poly(styrene sulfonate) (PSS) sodium salt standards of 246, 1000, 2000, 3400, 4200, 6000, 10 000, 15 000, 20 000 and 30 000 Da (Agilent, catalog numbers PSS‐PSS246 to PSS‐PSS30K). Each standard was dissolved in the GPC mobile phase (0.20 wt% NaN_3_ in DI water) to 1.0 mg mL^−1^, and injected (20 µL) in duplicate. Chromatography was performed on a PL‐Aquagel OH Mixed‐H column (300 × 7.5 mm, 8 µm) held at 40 °C with an Agilent 1260 pump (1.0 mL min^−1^) and UV detection at 280 nm. Retention volumes were converted to log M using a third‐order polynomial fit (*r* ^2^ > 0.999). Calibration details are provided in Table S5, and the calibration curve is shown in Figure S8. The resulting calibration was applied to all depolymerization and nanofiltration samples analyzed under identical conditions.

### 2.9 High‐Performance Liquid Chromatography (HPLC)

The quantification of individual monomer classes was performed on the same instrument fitted with two complementary columns. For aliphatic acid analysis, a Bio‐Rad Aminex HPX‐87H (300 mm × 7.8 mm) was used; 4 mM H_2_SO_4_, 0.60 mL min^−1^; column 60 °C; RI detector 35 °C. For aromatic products, an Agilent Eclipse Plus Phenyl‐Hexyl (250 mm × 4.6 mm, 5 µm) was used; gradient elution from 30 % to 100 % solvent B in 12 min (solvent A: 10 mM NH_4_OAc/0.07 % HCOOH in H_2_O, solvent B: 10 mM NH_4_OAc/0.07 % HCOOH in 90 % MeCN); 0.5–1.0 mL min^−1^; column 50 °C; DAD at 254, 280, and 310 nm. Samples were centrifuged, tenfold diluted with the initial mobile phase, filtered (0.20 µm PTFE), and analyzed in duplicate. Calibration curves (*R* ^2^ > 0.995) were prepared from authentic standards spanning from 0.01 to 1.0 g/L for each analyte. Resulting concentrations were used to compute yield (Equation S2) and rejection (Equation S4) values, which in turn underpin the separation‐ factor calculations discussed in the main article.

## 3 Results and Discussion

To evaluate the products of catalytic lignin depolymerization in detail, six reactions were conducted using poplar lignin (0.35 g) with different catalysts and conditions (Table 1), adapting protocols from our recent research on polyoxometalate (POM)‐ionic liquid systems [41, 44]. The present work focuses on membrane separation, and the catalysis experiments are used primarily to generate compositionally distinct depolymerized streams for evaluating downstream rGO membrane fractionation. POMs were chosen as benchmark redox catalysts for aqueous oxidative lignin depolymerization. H_2_O_2_ was included to intensify oxidation and probe how stronger radical chemistry shifts monomer/oligomer distributions. The POM/H_2_O_2_/O_2_ system promotes extensive oxidative de‐aromatization of lignin, consistent with our recent work [41] with the same POM catalyst. In that study, lignin conversion and breakdown were thoroughly studied to suggest depolymerization mechanisms and the formation of low‐MW oxidation products. While C─O and C─C bonds were cleaved, the extent of C─O bond cleavage (notably β─O─4) could be controlled, and uncontrolled condensation was limited. NaOH represents the conventional alkaline baseline (base‐promoted ether cleavage and lignin solubilization) and provides a practical reference point for industrially relevant “cooking”‐type conditions. GO was evaluated as a metal‐free carbocatalyst to test whether surface redox functionalities alone can drive measurable depolymerization under mild conditions. A non‐catalyst control establishes the contribution of thermal treatment and the reaction medium. Together, this matrix spans oxidative vs. alkaline regimes while keeping other variables constant, allowing catalyst‐driven changes in molecular weight distributions to be directly linked to membrane fractionation performance. Lignin conversion was determined from the recovered solids (Equation S1), and the corresponding conversion values and solid mass balances are summarized in Table S4. The GPC chromatograms of the reaction products are shown in Figure 2a. It is important to note that in GPC, higher MW species elute earlier (with shorter retention times), whereas lower MW species elute later. The native poplar lignin exhibited a sharp and narrow peak at a retention time of ~5.8 min, corresponding to higher MW polymers with minimal prior degradation. In contrast, depolymerization reactions catalyzed by polyoxometalates (POMs) (reactions 3, 4, and 5) yielded substantial shifts in the GPC profiles toward higher retention times (7–10 min), indicating a reduction in average MW and the formation of lower‐MW oligomers. The GO‐catalyzed (reaction 2) and non‐catalyzed control (reaction 1) cases resulted in minimal MW reduction. While GO materials are known to possess redox‐active surface functionalities [45], our results suggest that the intrinsic catalytic activity of GO alone is insufficient for significant lignin bond cleavage, likely due to the low surface radical density or limited substrate interaction.

**TABLE 1:** Summary of lignin depolymerization reaction conditions. All reactions were performed on 0.35 g of poplar lignin.

Reaction number	Catalyst	*T*, °C	*P*, bar	Pressurized gas	Reaction time, h	Additive
1	No catalyst (control)	140	10	O_2_	1	None
2	GO (0.1 g)	140	10	O_2_	1	None
3	POM (0.1 g)	140	10	O_2_	1	1 wt% H_2_O_2_
4	POM (0.1 g)	140	10	N_2_	1	1 wt% H_2_O_2_
5	POM (0.1 g)	140	10	O_2_	1	None
6	NaOH (0.1 g)	140	10	O_2_	1	None

**FIGURE 2:**
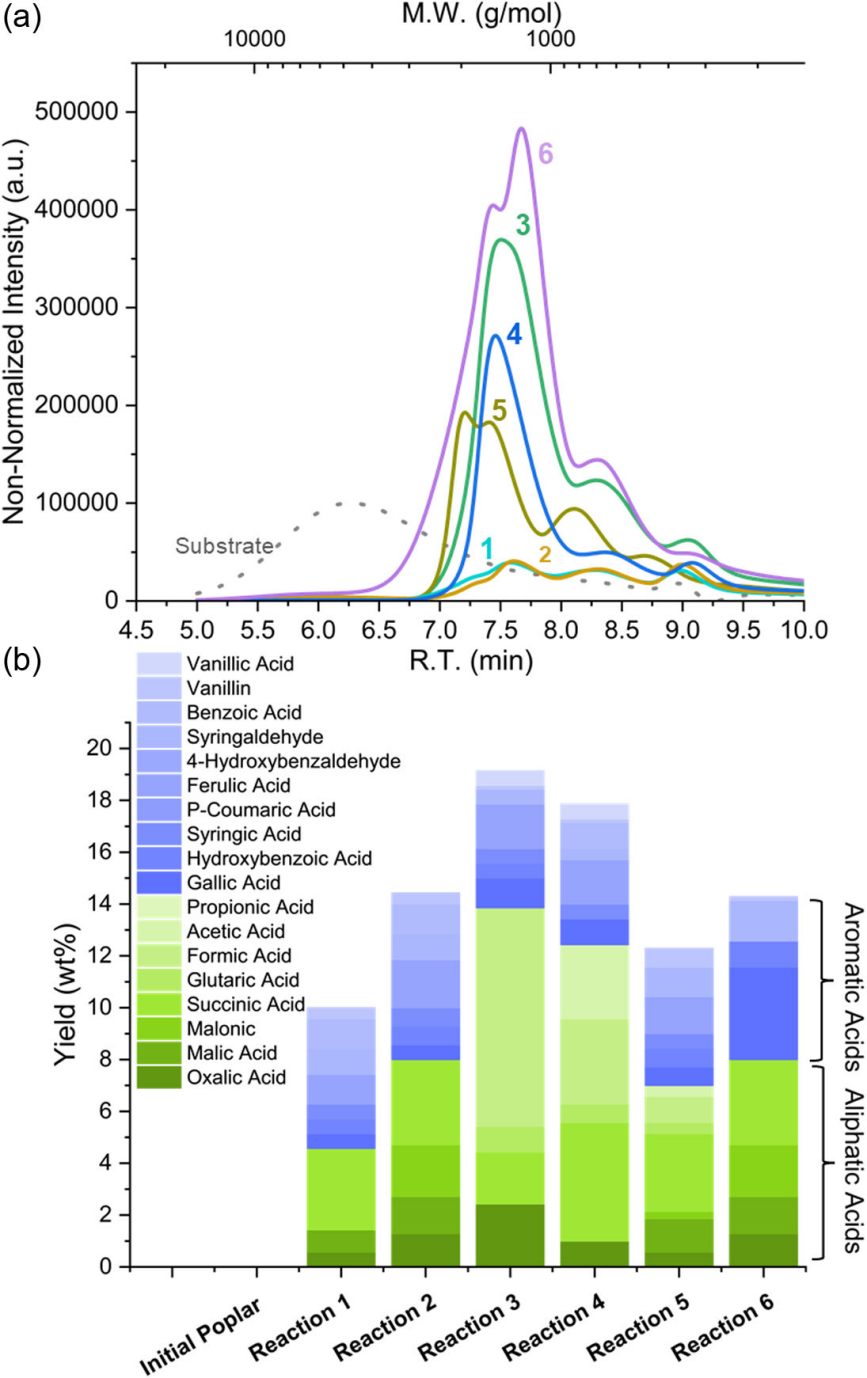
Lignin depolymerization results. (a) Non‐normalized GPC spectra of reactions 1–9 (dotted line: initial poplar lignin, intensity ×1000 for comparison due to low solubility in water) and (b) monomer yields of aliphatic and aromatic products.

Reaction 3, employing POM in combination with 1 wt% H_2_O_2_ as the additive under 10 bar of O_2_ pressure, demonstrated the most pronounced depolymerization. The resulting chromatogram revealed a significant decrease in high‐MW components and a marked shift toward low‐MW fragments, a trend mirrored in the HPLC‐quantified monomer yields computed from Equation S2 and reported in Table S1 (aliphatics) and Table S2 (aromatics), suggesting enhanced oxidative bond cleavage. This outcome underscores the synergistic role of oxidative additives in facilitating selective β─O─4 linkage cleavage, which is consistent with prior studies on radical‐mediated lignin fragmentation [46, 47]. Reaction 6, conducted with 0.1 g of NaOH, represents a conventional alkali “cooking” approach and showed only moderate depolymerization. The corresponding GPC profile retained a slight shoulder at a lower retention time (~6.0 min), suggesting the persistence of partially depolymerized high‐MW species. This observation implies that while NaOH facilitates hydrolytic bond scission, it lacks the oxidative strength to break the internal ether linkages. Importantly, the control of catalyst amount, lignin loading, and pressure across all reactions ensures that the observed variations arise predominantly from catalyst identity and additive presence. This highlights the greater efficacy of transition‐metal‐based redox catalysts (POM) over base‐catalyzed or carbon‐based systems for lignin depolymerization. This ranking is consistent with the expected mechanisms of lignin bond scission. The oxidative catalyst (POM in this study) enables selective β─O─4 ether cleavage as well as de‐aromatization of phenolic units through redox cycling and oxygen activation [41, 44], whereas GO lacks sufficient catalytic sites for electron transfer. NaOH promotes partial cleavage through base‐catalyzed hydrolysis but is insufficient alone for efficient delignification.

Based on the analysis of the HPLC results (Figure 2b) obtained using the Aminex HPX‐87H column for aliphatic acids and the phenyl hexyl column for aromatic compounds (more characterization protocols are shown in Supporting Information, and representative chromatograms are shown in Figure S2), the product profiles from various catalytic lignin depolymerization reactions reveal clear differences in catalytic efficiency and product selectivity. The POM‐catalyzed depolymerization in the presence of H_2_O_2_ under an O_2_ atmosphere yielded the highest total aliphatic acid content (14 wt%) and a notable aromatic product yield (5.3 wt%). This suggests that the oxidative environment introduced by H_2_O_2_ facilitates more effective C─C and C─O bond cleavage in lignin, promoting aliphatic acid formation. Conversely, the GO‐catalyzed depolymerization resulted in 8 wt% aliphatic acids and the highest aromatic product yield of 6.4 wt%, indicating that GO may favor pathways preserving aromatic core structures. The enhancement of the aliphatic acid yield by H_2_O_2_ was not accompanied by a corresponding increase in aromatic product formation, implying selective promotion of oxidative cleavage rather than aromatization or stabilization processes. Small aliphatic acids are formed through sequential oxidative fragmentation of lignin‐derived intermediates. Cleavage of ether linkages and side‐chain C─C bonds, particularly Cα─Cβ, generates oxygenated intermediates that can undergo further oxidation and partial aromatic‐ring opening to form LMW carboxylic acids [41, 48, 49]. Aromatic acids arise primarily from the oxidation of lignin‐derived aromatic alcohols and aldehydes while retaining the aromatic ring [15, 49].

The most abundant aliphatic products identified include formic and succinic acids, both key indicators of lignin fragmentation and side‐chain oxidation. For the aromatic fraction, gallic and ferulic acids were prominent, reflecting the partial preservation of lignin’s native aromatic units. Notably, reactions performed under N_2_ (e.g., POM‐N_2_ condition) produced lower aliphatic yields, underscoring the importance of oxidative gas–liquid equilibrium in driving depolymerization efficiency. These findings confirm that the combination of catalyst type, oxidizing environment, and additive presence critically governs the molecular distribution and yield of depolymerized lignin products.

Figure 3 depicts a representative PL‐Aquagel GPC trace of the water‐soluble fraction obtained after oxidative depolymerization and liquid–liquid partitioning. Calibration with polystyrene sulfonate sodium salt standards allows the chromatogram to be divided into three nominal molecular‐weight (MW) zones/windows that are subsequently used throughout this study for membrane performance metrics (yellow boundaries in Figure 3) [26, 30, 50]. For consistency, the boundaries are defined at the minima (at 800 and 1600 Da) between the three main peak regions. It is emphasized that GPC shows a continuous MW spectrum. Thus, any division into MW zones only implies an enriched concentration (and not 100% purity) of the assigned species in that zone. Zone I (≤ 800 Da) includes aromatic monomers (Figure S3) and other low‐MW products. Zone II (800 Da < MW ≤1600 Da) contains short lignin oligomers generated by β─O─4 cleavage. Zone III (MW >1600 Da) represents the high‐MW fraction of partially depolymerized lignin residues. These windows were used for integrated peak‐area quantification and to report membrane fractionation performance.

**FIGURE 3:**
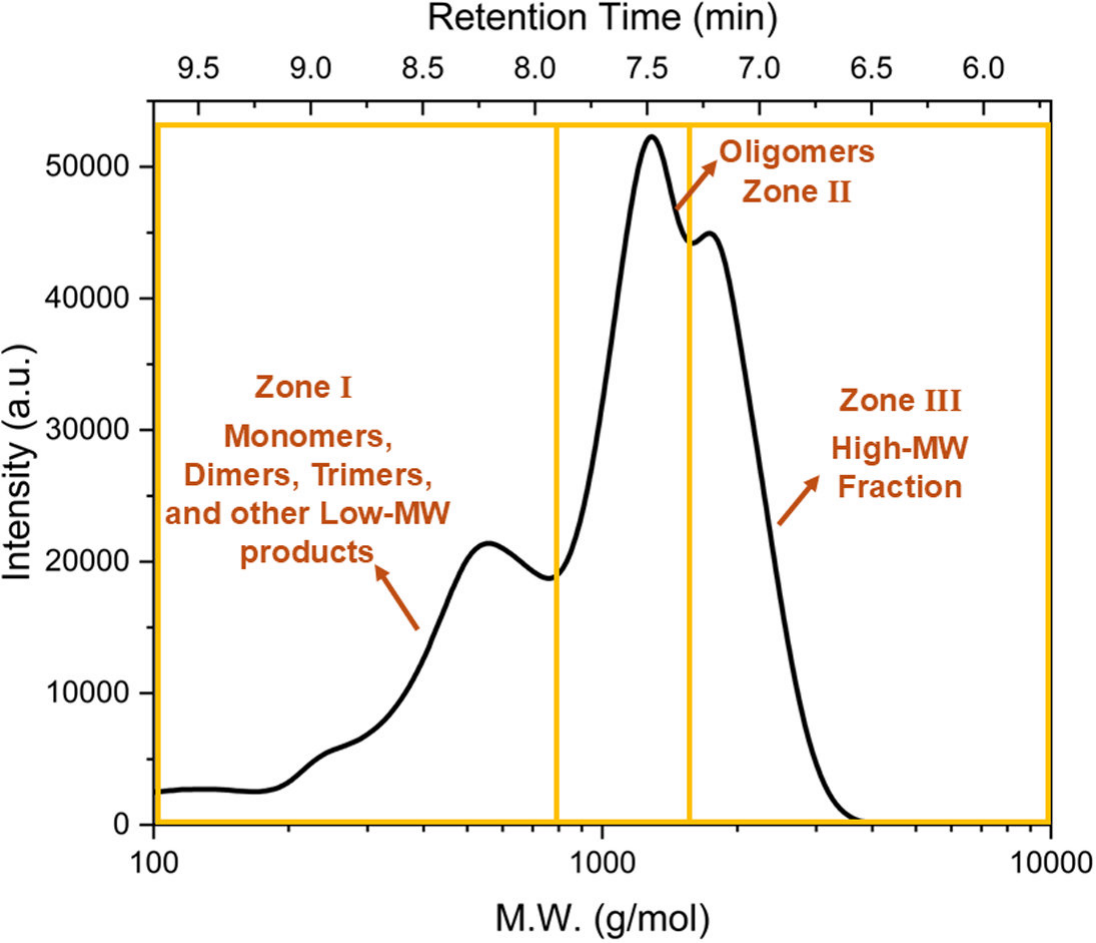
Representative aqueous GPC chromatogram of depolymerized‐lignin liquor (from reaction 2) illustrating operational apparent‐molecular‐weight windows: Zone I (LMW, ≤0.8 kDa), zone II (oligomeric, 0.8 < MW ≤1.6 kDa), and zone III (HMW/polymeric, MW >1.6 kDa).

Because PL‐Aquagel lacks the resolution to deconvolute individual monomers within zone I, absolute concentrations of aliphatic and aromatic acids are quantified by HPLC methods (Aminex HPX‐87H for carboxylates and phenyl‐hexyl for phenolics). The HPLC‐derived monomer concentrations were used for rejection and cut calculations specific to chemically valuable species, while GPC peak areas for zones I–III furnish the mass balances required to compute overall separation factors (Equation S6) for LMW products and oligomers compared to the HMW fraction. Peak deconvolution/integration is shown in Figure S4. GPC provides the apparent MW distribution of the overall soluble product mixture based on the hydrodynamic size. In the low‐MW region, the PL‐Aquagel column has limited resolving power, and multiple monomeric species co‐elute within a relatively narrow retention‐time window. Hence, changes in individual monomer concentrations may not produce a clearly distinguishable change in the overall GPC envelope. Moreover, the GPC signal is a detector response across the full MW distribution and is not itself a direct measurement of the monomer yield. In contrast, calibrated HPLC methods quantify compound‐specific monomer yields. Thus, the similar GPC profiles of reactions 1 and 2 indicate only a limited change in the bulk MW distribution, whereas HPLC resolves an increased abundance of specific aliphatic and aromatic monomers within the low‐MW fraction. This dual‐technique workflow ensures that both chemically specific (monomer) and molecular‐weight‐specific (oligomer/HMW) performances are captured with high fidelity in the subsequent membrane fractionation analysis.

Initially, the six poplar‐lignin liquors produced under distinct oxidative conditions (reactions 1–6) were all subjected to nanofiltration through the same PVDF‐supported rGO membrane (rGO‐PVDF, nominal MWCO ≈ 470 Da, as shown in Figure S1, Δ*p* = 10–30 bar, 25 °C, 190 mL min^−1^). Bare PVDF exhibited no measurable rejection of the lignin‐derived solute fractions under the same operating conditions (Table S7), confirming that the observed molecular‐weight selectivity originates from the rGO laminate rather than from the porous support. Beyond the single‐point MWCO metric, the rGO membrane exhibits rejection behavior consistent with an ensemble of sub‐nanometer slit pores within the established pore‐size‐distribution framework. The rGO membrane is characterized by an effective pore radius of *a* ^*^ = 0.54 ± 0.12 nm [42]. GPC traces displaying two pH‐distinct examples are shown in Figure 4a,b. Irrespective of the catalytic history, the membrane almost quantitatively separates the broad high‐MW peak (>1.6 kDa) characteristic of crude depolymerizates, confirming that steric exclusion is essentially pH‐independent. In contrast, the fate of chemically valuable low‐MW species proves highly pH‐ sensitive (Figure 4c). Our previous work has established the isolation of steric molecular sieving from charge‐mediated rejection in rGO membranes using charge‐neutral molecular probes (glucose, xylose, and maltose), with their rejections directly correlated with interlayer microstructure and effective pore‐size distributions [42, 51, 52]. Consistent with this framework, the persistent ≥90% rejection of the >1.6 kDa fraction across the present pH range indicates no appreciable shift in the effective steric cut‐off; thus, the pronounced pH dependence of low‐MW carboxylate rejection is attributed predominantly to ionization‐dependent electrostatic exclusion. Minor pH‐induced changes in gallery hydration cannot be completely excluded. In the most acidic liquor (e.g., pH 2.5), carboxylic acids are largely protonated, while phenolics are only weakly dissociated; their near‐neutral charge permits facile passage through the negatively charged slits, giving the highest separation factors for both aliphatic (≈ 6) and aromatic (≈ 5) monomers, with a still‐modest value (≈ 2) for 800–1.6 kDa oligomers that experience steric drag. When the liquor becomes mildly acidic (e.g., pH 5.2), first‐stage de‐protonation of di‐ and tri‐acids increases anionic charge and Donnan repulsion [53, 54], roughly halving the aliphatic acid separation factor, whereas predominantly neutral phenolics are less affected. Approaching neutrality (e.g., pH 6.6), further de‐protonation suppresses acid transport to such an extent that the oligomer separation factor overtakes that of every monomer class, marking a transition from size‐sieving to charge‐dominated selectivity. Under alkaline conditions (e.g., pH 9.2), near‐complete ionization of all carboxylates maximizes electrostatic rejection; the total‐monomer separation factor collapses to about unity and the aliphatic‐acid value falls below one, signifying that charged monomers are now co‐retained with oligomers, while the membrane still rejects more than 90 % of the >1.6 kDa fraction, attesting to its structural stability at high pH. Catalyst identity and trans‐membrane pressure exert only a secondary influence, and feed pH is the dominant factor for modulating monomer/oligomer partitioning across the graphene‐oxide membrane. Acidic feeds operate in a steric‐control regime that enriches monomers in the permeate, whereas alkaline feeds enter an electrostatic‐control regime that co‐retains acids and oligomers. A representative visual comparison of the permeate and retentate fractions is provided in Figure S7. This mechanistic understanding guided the choice of the monomer‐rich, low‐pH Reaction 3 liquor for the integrated diafiltration study described in the long‐term fractionation study, where continuous dilution further amplifies size‐ and charge‐based discrimination while purging recalcitrant high‐MW fragments from the loop.

**FIGURE 4:**
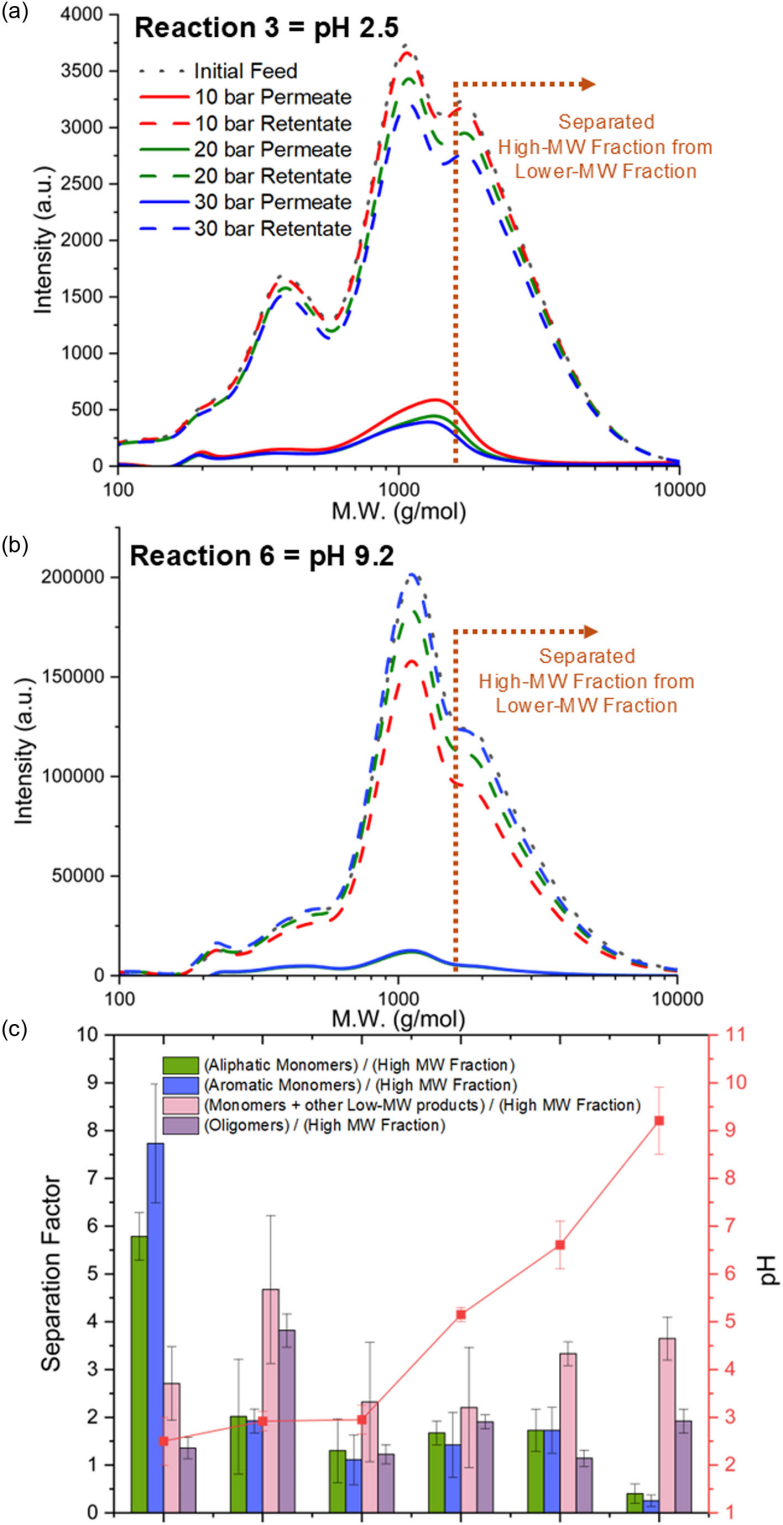
Separation of lignin depolymerizates from diverse catalytic feed streams. (a, b) GPC traces of permeate (solid) and retentate (dashed). (c) Separation factors for aliphatic acids, aromatic monomers, low‐MW products (≤ 1 kDa), and oligomers (1–1.6 kDa) relative to the high‐MW fraction (> 1.6 kDa) as a function of feed pH.

To better understand the membrane separation effects independent of catalyst‐specific matrix effects, we prepared three model feeds that replicate the monomer distribution of the high‐yield POM/H_2_O_2_ run (reaction 3). These feeds comprised (i) aliphatic acid only, (ii) binary oxalic‐ferulic pairs, and (iii) the full aliphatic + aromatic cocktail. All experiments were performed with the same rGO layer on a PVDF support (MWCO ≈ 470 Da) operated in a cross‐flow nanofiltration cell at 25 °C, 190 mL min^−1^, and trans‐membrane pressures of 10‍–‍30 bar. The completed list of product compositions used in the model solution experiments is provided in Table S3. We evaluated the effects of pH, solute concentration, temperature, and aromaticity on the separation efficiency using rGO membranes. Figure 5a demonstrates that the rGO/PVDF membrane discriminates aliphatic acids (formic–glutaric, 46–132 g mol^−1^) almost exclusively through electrostatic interactions. At the native reaction pH of 2.5 (reaction 3’s original pH), the acids are >95 % protonated, charge repulsion is negligible, and transport is governed by molecular sieving. The ensemble rejection was therefore <20 %. Raising the feed to pH 6 induces the first de‐protonation step (pK_a1_ ≈ 3–5), imparting a single negative charge to each molecule. The resulting Donnan potential across the charged galleries increases the average rejection threefold. Further alkalization to pH 11 drives complete de‐protonation, maximizing surface‐solute electrostatic repulsion and pushing the rejection to a pressure‐invariant plateau of 70%–80 % (Figure 5a). The loss of trans‐membrane pressure dependence in this highly charged regime confirms that charge exclusion, rather than molecular size, controls carboxylate partitioning through the rGO nanochannels.

**FIGURE 5:**
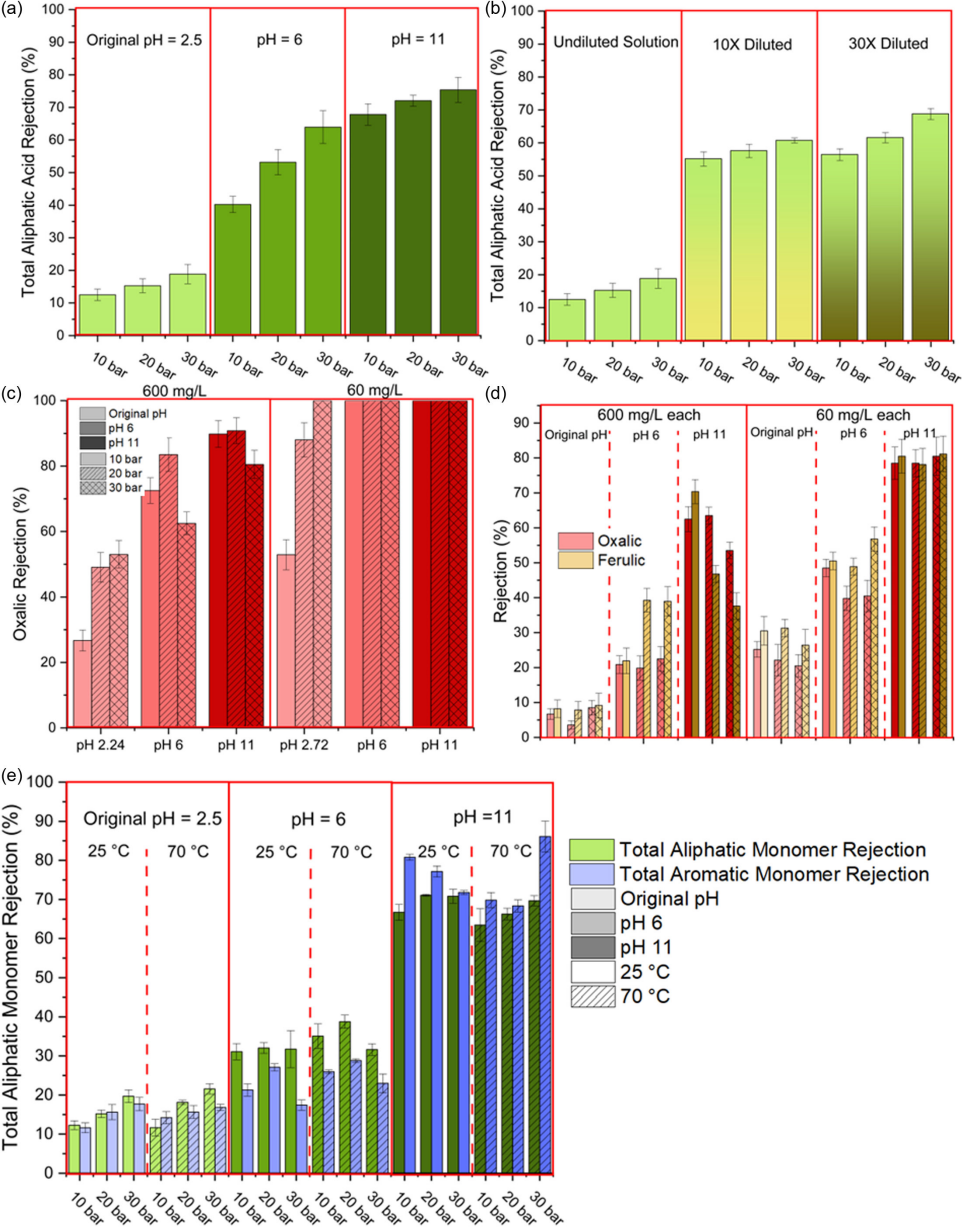
Membrane rejection profiles from rGO/PVDF membrane for lignin‐derived model mixtures: (a) Effect of pH on aliphatic acid mixture; (b) effect of dilution on aliphatic acid mixture; (c) oxalic acid rejection at different pH and concentration; (d) comparison of oxalic versus ferulic rejection at different pH and concentration; and (e) combined effect of pH, temperature, and pressure on mixed aliphatic and aromatic synthetic mixture approximating reaction 3.

Beyond pH, the solute concentration had a notable effect on rejection behavior. Dilution (10X and 30X) increased the observed acid rejections, consistent with reduced concentration polarization and lower ionic‐strength screening, which together strengthen the effective steric/electrostatic selectivity of nanofiltration [55, 56]. Dilution also likely decreases the competitive binding of solutes to membrane pores and active sites, promoting more selective transport. In contrast, the undiluted solution showed the lowest rejection at pH 2.5, which, while indicating reduced electrostatic exclusion due to protonation and high ionic strength, is beneficial for maximizing monomer transmission into the permeate. This highlights the utility of operating under non‐diluted conditions when the separation goal is to effectively distinguish and recover monomeric products from higher‐molecular‐weight oligomers. For the aliphatic suite (Figure 5a), rejection increased monotonically with pH because successive de‐protonation augments the dianionic carboxylate charge, enhancing Donnan exclusion [53, 54]. Oxalic acid, the smallest and most highly charged acid (pK_a1_ ≈ 1.3 and pK_a2_ ≈ 4.3), reached ≈100 % rejection at pH 11 but fell below 20 % at pH 2.5 when largely neutral. The same solute, when present at 600 mg/L, was rejected by 15–20 percentage points less than at 60 mg/L, indicating concentration polarization and site saturation of charge‐compensating regions on the graphene oxide surface. Although increased transmembrane pressure from 10 to 30 bar produced a slight improvement in rejection, electrostatic forces remain the dominant separation mechanism. These findings validate the use of oxalic acid as a proxy molecule for assessing membrane performance, especially under conditions relevant to lignin depolymerization streams. Introducing an equal amount of ferulic acid (MW 194 g/mol; aromatic pKa ≈ 4.6) fundamentally altered this picture (Figure 5d). At pH 2.5, oxalic acid rejection dropped by up to 25 %, whereas ferulic acid rejection rose. We ascribe the divergence to π–π and CH–π stacking between the electron‐rich ferulate ring and the sp^2^ graphene domains. Even at pH 11, oxalate rejection falls by 10%–15 % in the oxalate + ferulate pair because π‐rich ferulate both partially occludes the rGO nano‐slits and neutralizes slit‐wall charge, weakening the Donnan barrier that would otherwise exclude the smaller dianion. Transient interaction of ferulate on the basal planes narrows the effective pore cross‐section and locally (i) screens the slit‐wall charge, easing the passage of the smaller oxalate and (ii) traps the bulky aromatic itself, raising its apparent rejection. This competitive interaction mechanism foreshadows the behavior of full multi‐solute feeds, in which an ensemble of aromatics collectively modulates the charge and steric landscapes within the nanochannels.

Synthetic mixtures approximating the composition of reaction 3 were used to examine how mixed solute systems behave under different pH and temperature conditions (Figure 5e). At pH 11, total rejection of aliphatic and aromatic components reached ~75%, whereas at pH 2.5, rejection dropped sharply to <20%. However, the rejection efficiency in mixed systems was consistently lower than that in aliphatic‐only systems at equivalent pH. These results indicate that aromatic species actively disrupt the selectivity of the membrane, possibly through transient π–π and CH–π interactions or by altering local charge environments near the membrane surface. Despite the addition of aromatic content, temperature had little influence on the separation performance. Increasing the process temperature from 25 to 70 °C had a negligible effect on rejection, confirming the thermal robustness of rGO membranes and their potential compatibility with industrial‐scale lignin depolymerization processes.

Next, we assessed how the polymeric support influences the structural integrity and separation performance of rGO membranes in depolymerized lignin streams. PVDF substrates were used for fabricating multiple membranes for extensive separation measurements reported above, consistent with our recent work on organic solvent nanofiltration with PVDF‐supported rGO membranes [43]. However, PES is known to provide higher long‐term stability and structural rigidity in aqueous lignin‐rich streams, consistent with our prior development of PES‐supported rGO membranes for black liquor processing [28]. Photographs of membrane coupons and substrate details are shown in Figure S5. Figure 6a,b summarize the influence of the two polymeric supports on the fractionation of the POM‐catalyzed + H_2_O_2_ depolymerization liquor using reduced graphene oxide (rGO) nanofiltration laminates as the selective layer. For the undiluted feed, both rGO/PVDF and rGO/PES membranes efficiently remove the broad high‐MW (>1600 Da) fraction; however, the rGO/PES membrane removes the high‐MW fraction more cleanly, as evidenced by the lower permeate intensity above ~1 kDa and by separation factors that reach ~12 for total monomers/HMW at 30 bar, compared with ~7 for the PVDF analog (Figure 6b and Table S6). After diluting the feed liquor by 30X, rGO/PES still outperforms rGO/PVDF, with monomers/high‐MW separation factors climbing to ~13 and oligomer rejection >98%. As a result, we selected rGO/PES membranes for further investigation in long‐term continuous fractionation experiments that started from the non‐diluted feed. We used diafiltration for the complete, long‐term separation of low‐ and high‐MW fractions.

**FIGURE 6:**
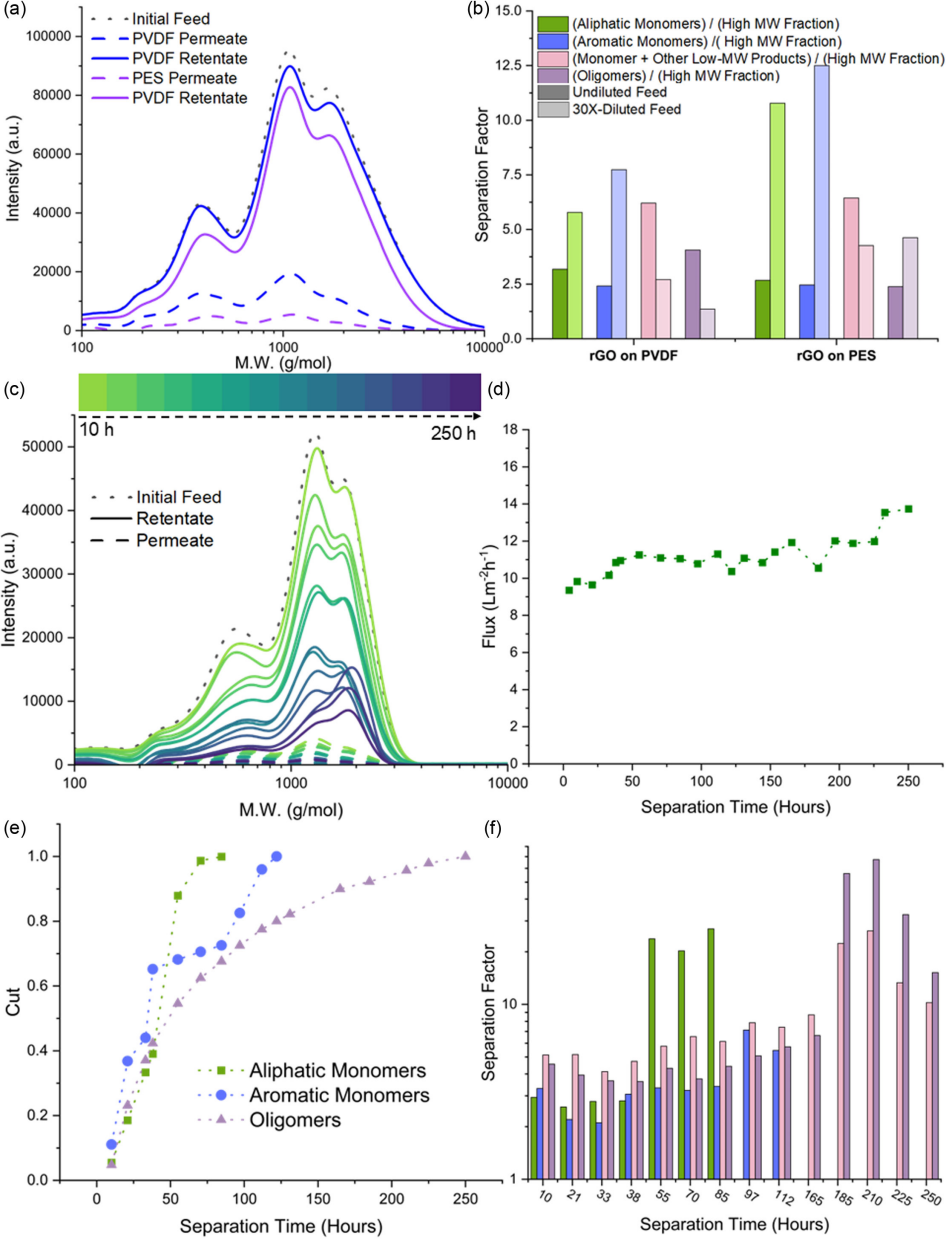
Nanofiltration and diafiltration of reaction‐3 liquor using rGO membranes. Nanofiltration (NF; batch crossflow, no dilution): (a) GPC traces and (b) separation factors for PVDF‐ and PES‐supported rGO. Diafiltration (DF; continuous crossflow with periodic water addition to maintain volume): (c) Time‐resolved GPC spectra over 250 h using PES‐supported rGO and (d) flux profile. (e) Cumulative monomer cuts. (f) Time‐resolved separation factors. Conditions: 30 bar, 190 mL/min, 25 °C.

Figure 6c–f summarizes a 250 h continuous diafiltration of an undiluted oxidative‐depolymerized lignin liquor through an rGO/PES nanofiltration membrane (30 bar, 190 mL min^−1^, 25 °C). Time‐resolved GPC spectra (Figure 6c) show a monotonic depletion of high‐MW species (MW > 1600 Da) in the permeate and their concurrent enrichment in the retentate, confirming sustained size‐selective exclusion. Flux remained stable at 11 ± 1 L m^−2^ h^−1^ (Figure 6d) and even rose slightly after each water addition interval, reflecting the progressive reduction in feed viscosity and concentration polarization rather than membrane compaction or fouling. Integration of HPLC‐quantified monomer yields produced cumulative cut curves (Figure 6e). Aliphatic acids reached complete recovery (cut ≈ 1.0) after ~85 h, while aromatic monomers required ~112 h, consistent with their higher single‐solute rejection attributed to π–π interactions with the rGO basal planes. Cumulative cuts were calculated using Equation S7. The corresponding separation factors (Figure 6f) highlight these dynamics: aliphatic monomers rose steadily and plateaued at values of ~10–12 relative to the HMW fraction, and aromatic monomers increased more slowly before stabilizing at ~7–9 once the aromatic pool was exhausted, while oligomers maintained modest values of ~3 in the early stages but increased to >15–17 after ~185 h as the retentate became enriched almost exclusively in oligomeric and polymeric species. Importantly, there was no detectable loss of performance after ten days of continuous operation, underscoring the robustness of rGO membranes in complex lignin streams.

Operationally, the experiment delineates an optimal processing window: early diafiltration (≤120 h) maximizes monomer harvest, whereas extended operation (>180 h) purges residual oligomers, delivering an HMW‐rich retentate. Downstream, the monomer‐enriched permeate can be polished with a tighter nanofiltration membrane to decouple the oligomer from dimers, trimers, and acids/phenolics, enabling a two‐cascade scheme that furnishes three value‐defined lignin streams without solvent‐intensive steps. By sustaining stable flux and reproducible separation factors over long‐term operation, rGO membranes generate well‐defined molecular‐weight group streams that can be directed to diverse downstream applications in both lignin conversion and material utilization. The rGO membrane demonstrated exceptional stability across the full operating envelope tested, with feed pH 2.5–11, temperatures of 25 and 70 °C, and transmembrane pressures of 10–30 bar. Within this range, pure water permeance at 25 °C and 10 bar varied by <3% after 20 consecutive pressure/temperature cycles, underscoring the chemical and mechanical robustness of both the selective rGO laminate and the underlying polymer supports. Extended diafiltration of the LMW‐rich Reaction‐3 liquor (30 bar, 190 mL/min, pH ≈ 2.5) over 250 h established a stability index, SI = 1 − Δ*J*/*J*_0_, that remained close to unity under acidic conditions and as high as 0.9 under alkaline operation (pH ≈ 9.2), with a negligible change in selectivity (ΔSF < 5%).

The above detailed membrane nanofiltration (and diafiltration) measurements were also corroborated by structural and chemical characterizations before and after the long‐term separation experiments. The XPS survey (Figure 7a) revealed nearly identical profiles of C and O, with only a slight increase in the C:O ratio from ≈15 to ≈19 after operation. This shift is attributed to weak and reversible adsorption of aromatic compounds onto the graphitic basal planes rather than to permanent oxidative loss or structural rearrangement of the laminate. High‐resolution C 1s spectra (Figures 7b and S10), deconvoluted using Gaussian peak fitting, further confirmed the chemical durability of the membrane. The spectra were resolved into contributions from C─C/C─H (284.6 eV), C─O (286.2 eV), C═O (287.0 eV), and O─C═O (289.0 eV) bonding environments [37, 42, 57] (±0.5 eV variation is within the accepted XPS uncertainty). Quantitative peak integration showed that after 250 h of acidic diafiltration, the dominant C─C/C─H peak increased slightly (+1.9%), consistent with physisorption of aromatic lignin fragments, while the concomitant drop in C─O (−10.5%) and C═O (−48.8%) signals indicates that adsorbed species are not forming new oxide functionalities on the carbon lattice (i.e., no oxidative attack on rGO). In contrast, the O─C═O contribution increased by 150.4%, suggesting surface enrichment by carboxyl‐containing lignin oligomers. Importantly, these shifts were subtle and fully reversible, with no evidence of irreversible oxidative degradation. Morphological analysis by SEM (Figure 7c,d) reinforced this conclusion. Cross‐sectional SEM confirmed intact membrane thickness and adhesion to the polymer support, with no evidence of obvious foulant deposit layers or morphological changes. To further test the robustness and foulant cleaning of the membrane, we performed an aggressive alkaline cleaning test (1 N NaOH at 25 °C under 30 bar for 12 h) that we previously used [43]. The pure‐water flux recovered to the same level as the initial water flux (Figure S11), indicating that fouling was highly reversible. This is useful information to inform further selection and optimization of periodic chemical cleaning and recovery protocols [33, 58, 59] when planning larger‐scale piloting. Taken together, the present findings demonstrate that rGO membranes combine pH‐responsive selectivity with structural and chemical endurance. Their ability to tolerate depolymerized lignin streams, elevated temperatures, and sustained 30‐bar operation while resisting fouling, compaction, and degradation establishes them as robust candidates for integration into continuous catalytic depolymerization and separation schemes to realize lignin valorization.

**FIGURE 7:**
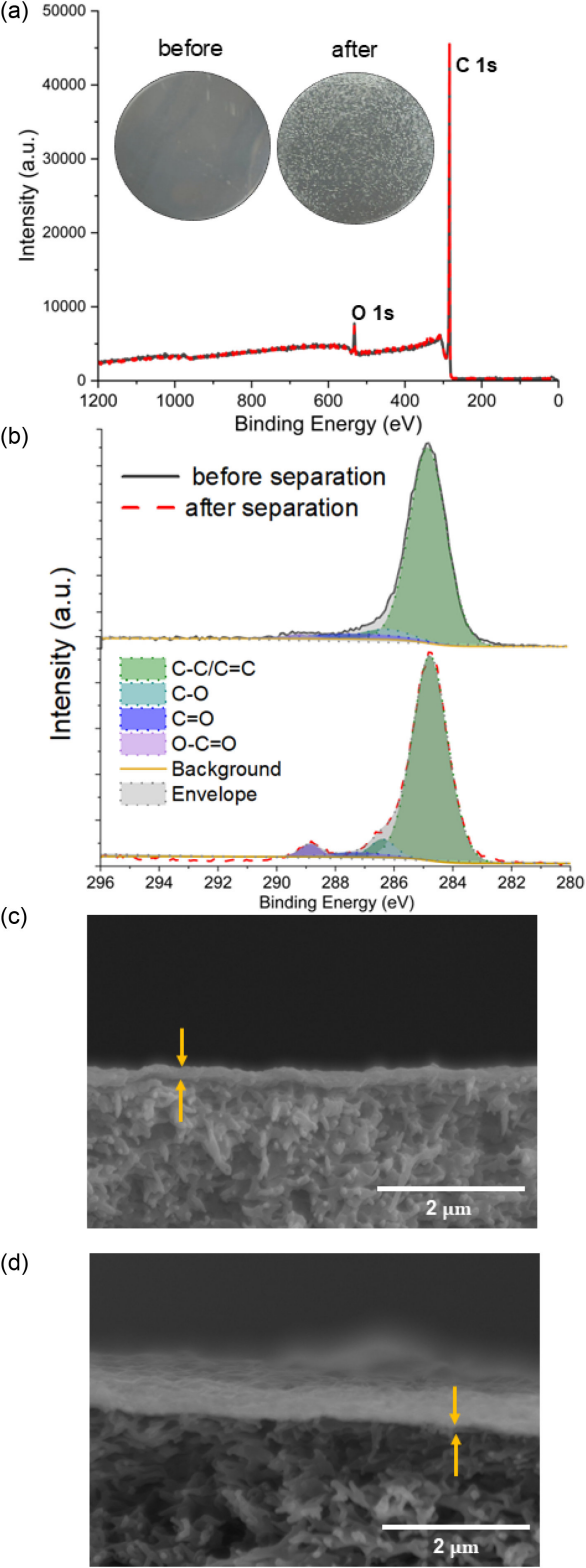
Structural and chemical stability of rGO/PES membranes before and after 250 h lignin fractionation. (a) XPS survey spectra with optical images of 47 mm diameter coupons (inset); (b) High‐resolution C 1s XPS spectra; (c) Cross‐sectional SEM of pristine membrane; (d) Cross‐sectional SEM after separation (with yellow arrows marking the rGO laminate on PES).

## 4 Conclusion

This work demonstrates that the rGO nanofiltration membrane enables aqueous, energy‐efficient fractionation of oxidatively depolymerized lignin into a monomer‐enriched permeate and a high‐MW retentate suitable for recycling or alternative valorization. Regardless of catalyst history, rGO membranes robustly rejected ≥90% of the >1.6 kDa fraction, confirming that the removal of recalcitrant oligomers/polymers is primarily governed by steric exclusion through sub‐nanometer slit pathways. In contrast, feed pH emerged as the dominant lever for tuning LMW transmission: acidic liquors favored monomer passage (steric control), whereas alkaline liquors promoted electrostatic exclusion, co‐retaining ionized carboxylates with oligomers (charge control). Model‐feed experiments further clarified how dilution and aromatic co‐solutes influence apparent selectivity through concentration polarization, ionic‐strength screening, and competitive interactions in graphitic nanochannels. Importantly, long‐term diafiltration of a representative monomer‐rich liquor sustained stable flux and cut‐off over 250 h, enabling quantitative monomer recovery while concentrating high‐MW residues, validating membrane durability under realistic, complex feeds. Collectively, these findings provide practical design rules (pH control, dilution strategy, and support selection) for integrating rGO membrane nanofiltration into continuous lignin‐upgrading workflows that require MW‐defined product streams.

## Author Contributions

This work was conceived by S.N., H.C., and B.A.S. Experimental work was performed by N.Y., D.M., Y.H., M.S., and S.S. Data analysis, manuscript drafting, and manuscript editing were primarily performed by N.Y., H.C., and S.N., with inputs from all authors.

## Funding

This work was supported by the U.S. Department of Energy (DE‐AC02‐05CH11231 and DE‐NA0003525) and the National Science Foundation (ECCS‐2025462).

## Conflicts of Interest

B.A.S. has a financial interest in Illium Technologies, Caribou Biofuels, and Erg Bio. N.Y., B.A.S., H.C., and S.N. are named inventors on at least one related patent application. The other authors declare no conflicts of interest.

## Supporting information

Supplementary Material

## Data Availability

The data that support the findings of this study are available from the corresponding author upon reasonable request.
