# Supplementary material for: Graphene Oxide Membranes for Recovery of Catalytically Depolymerized Lignin Monomers and Oligomers
Source: ChemSusChem. 2026 Oct 20;19(20):e71123. doi: 10.1002/cssc.71123 (PMC13681734; doi:10.1002/cssc.71123)
Supplement: Supplementary file 1 [file CSSC-19-e71123-s001.pdf]

Supporting Information

for

Graphene Oxide Membranes for Recovery of Catalytically Depolymerized Lignin Monomers and Oligomers

Natechanok Yutthasaksunthorn[1,3], Dhirendra Mishra[3], Yinglei Han[3], Muskan Sonker[1], Scott A. Sinquefield[2], Blake A. Simmons[3,4], Hemant Choudhary[3,5], and Sankar Nair[1,2] *

[1]*School of Chemical & Biomolecular Engineering, Georgia Institute of Technology, Atlanta, GA 30332, USA*

[2]*Renewable Bioproducts Institute, Georgia Institute of Technology, Atlanta, GA, 30332, USA*

[3]*Deconstruction Division, Joint BioEnergy Institute, Lawrence Berkeley National Laboratory, Berkeley, CA 94720, USA*

[4]*Biological Systems and Engineering Division, Lawrence Berkeley National Laboratory, Berkeley, CA 94720, USA*

[5]*Department of Bioresource and Environmental Security, Sandia National Laboratories, Livermore, CA 94550, USA*

* Corresponding author: sankar.nair@chbe.gatech.edu

Number of pages: 12

Number of supporting tables: 7

Number of supporting figures: 11

Experimental Methods

Metrics for Evaluating Lignin Depolymerization and Membrane Performance

All catalytic depolymerization experiments were evaluated on a mass basis, and the characterized intensities were normalized to compare the shift of the molecular weight in GPC. HPLC-quantified liquid products were included in the material balance; The quantities reported throughout the manuscript were computed as follows.

Lignin Conversion

A gravimetric approach was adopted because the polymeric residue contains incompletely solubilized and re-condensed fragments that are chemically heterogeneous but analytically inseparable. After reaction, the slurry was filtered (0.2 µm PTFE) and the solid cake was washed and vacuum-dried (60 °C, 12 h) to constant mass.

Conversion was then obtained from

$$X_{\boldsymbol{lignin}}(\%) = \frac{m_0 - m_{res}}{m_0} \times 100 \qquad \text{(Eq S1)}$$

where m_0 = initial poplar lignin mass (which is 0.35 g in all reactions for this study)

m_{res} = dry mass of the recovered solid residue

Monomer Yield

Each soluble product *i* (aliphatic acid or aromatic compound) was quantified by HPLC and reported as a weight yield relative to the starting lignin (7 g/L in this study):

$$Y_{\boldsymbol{i}}(\%) = \frac{m_i}{m_0} \times 100 \qquad \text{(Eq S2)}$$

where m_i = the mass of product *i* in each depolymerization product stream/ permeate/ retentate

Response-factor-corrected peak areas were converted to mass using calibration curves ($R^2 > 0.999$).

Membrane Fractionation Metrics

To quantitatively assess the solvent transport behavior, we report both flux (J, L·m^{-2}·h^{-1}) and permeance ($J/\Delta P$, L·m^{-2}·h^{-1}·bar^{-1}) in the subsequent discussion. The flux directly measures the throughput at a particular transmembrane pressure (TMP), whereas the permeance normalizes the flux with respect to the TMP. The two quantities thus allow more facile comparisons across different membranes, solvents, and measurement methods.

$$\text{Flux} \quad (J) = \frac{\Delta m}{A\,\rho\,\Delta t} \qquad \text{(Eq S3)}$$

where Δm is the mass of the permeate collected at each time (Δt), A is the effective area of the membrane, and ρ is the density of the permeate.

Membrane performance is usually expressed in terms of rejection (R_i), which quantifies how effectively a given solute is retained. This metric works well for solute–solvent operations, e.g., desalination or solvent-based resource recovery where the goal is to keep a contaminant out of the permeate. In lignin depolymerization product processing, however, the task is to discriminate between two solute classes rather than between solute and solvent. In this case, the parameter of greatest relevance is the separation factor (SF), defined as the ratio of permeabilities (or, equivalently, $1 - R_i$) of the two species (Eq 4). SF directly predicts the relative enrichment of each fraction and therefore provides a clearer measure of solute–solute selectivity than rejection alone.

$$Rejection\ (R_i) = \frac{C_i^r - C_i^p}{C_i^r} = 1 - \frac{C_i^p}{C_i^r} \qquad \text{(Eq S4)}$$

where C_i^p and C_i^r are the permeate and retentate sample concentrations.

Separation factor

The membrane's ability to discriminate between two product classes a and b is captured by the separation factor.

$$Separation\ Factor\ (SF) = \frac{\left(\frac{C_i}{C_j}\right)_{perm}}{\left(\frac{C_i}{C_j}\right)_{reten}} = \frac{\left(\frac{C_{perm}}{C_{ret}}\right)_i}{\left(\frac{C_{perm}}{C_{ret}}\right)_j} = \frac{1-Rej_i}{1-Rej_j} \qquad \text{(Eq S5)}$$

(Compound Group) Separation Factor

$$\frac{\left(\frac{C_{perm}}{C_{ret}}\right)_i}{\left(\frac{C_{perm}}{C_{ret}}\right)_j} = \frac{\left(\frac{area_{perm}}{area_{ret}}\right)_i}{\left(\frac{area_{perm}}{area_{ret}}\right)_j} \qquad \text{(Eq S6)}$$

Values > 1 signify preferential passage of class i over j. In this study SFs are reported for

(i) aliphatic monomers / HMW fraction,

(ii) aromatic monomers / HMW, and

(iii) oligomers / HMW, where "HMW" denotes material with MW > 1.6 kDa.

Cut (Cumulative Transmission)

During diafiltration, the bulk feed composition evolves; therefore, the cumulative cut of a solute group/ species at any time is preferred:

$$Cut = \frac{Targeted\ Molecules\ (Compound\ Group)\ in\ permeate}{Initial\ Amount\ of\ Targeted\ Molecules\ (Compound\ Group)\ in\ feed} \qquad \text{(Eq S7)}$$

This ratio expresses the mass of *compound/ species group* removed relative to the initial amount and approaches unity when the group is fully depleted from the retentate.

Supporting Tables

Table S1. Concentrations of water-soluble aliphatic monomers obtained from poplar-lignin depolymerization reactions 1–8 and the corresponding product yields.

Aliphatic Monomer / Reaction Number	Oxalic Acid (g/L)	Malic Acid (g/L)	Malonic Acid (g/L)	Succinic Acid (g/L)	Glutaric Acid (g/L)	Formic Acid (g/L)	Acetic Acid (g/L)	Propionic Acid (g/L)	Total Aliphatic Monomer Concentration (g/L)	**Aliphatic Monomer Product Yield (wt.%)**	S.D.
**Initial Poplar**	0.00	0.00	0.00	0.00	0.00	0.00	0.00	0.00			
**Reaction 1**	0.04	0.06	0.00	0.22	0.00	0.00	0.00	0.00	0.33	**4.72**	2.42
**Reaction 2**	0.09	0.10	0.14	0.23	0.00	0.00	0.00	0.00	0.55	**7.91**	2.15
**Reaction 3**	0.17	0.00	0.00	0.14	0.07	0.59	0.00	0.00	0.97	**13.89**	3.11
**Reaction 4**	0.07	0.00	0.00	0.32	0.05	0.23	0.20	0.00	0.87	**12.47**	1.89
**Reaction 5**	0.04	0.09	0.02	0.21	0.03	0.07	0.03	0.00	0.50	**7.14**	1.68
**Reaction 6**	0.09	0.10	0.14	0.23	0.00	0.00	0.00	0.00	0.55	**7.91**	2.08

Table S2. Concentrations of water-soluble aromatic monomers obtained from poplar-lignin depolymerization reactions 1–8 and the corresponding product yield

Aromatic Monomer / Reaction Number	Gallic Acid (g/L)	HBA Hydroxybenzoic Acid (g/L)	Syringic Acid (g/L)	P-Coumaric Acid (g/L)	Ferulic Acid (g/L)	4-Hydroxybenzaldehyde (g/L)	Syringaldehyde (g/L)	Benzoic acid (g/L)	Vanillin (g/L)	Vanillic Acid (g/L)	Total Aromatic Monomer Concentration (g/L)	**Aromatic Monomer Product Yield (wt.%)**	S.D.
**Initial Poplar**	0.00	0.00	0.00	0.00	0.00	0.00	0.00	0.00	0.00	0.00			
**Reaction 1**	0.04	0.04	0.04	0.00	0.08	0.00	0.07	0.08	0.03	0.00	0.39	**5.53**	2.1
**Reaction 2**	0.04	0.05	0.05	0.00	0.13	0.00	0.07	0.08	0.03	0.00	0.45	**6.43**	1.92
**Reaction 3**	0.08	0.04	0.04	0.00	0.12	0.00	0.04	0.00	0.01	0.04	0.37	**5.30**	2.05
**Reaction 4**	0.07	0.00	0.04	0.00	0.12	0.00	0.03	0.07	0.01	0.04	0.38	**5.40**	1.21
**Reaction 5**	0.05	0.05	0.04	0.00	0.10	0.00	0.08	0.00	0.05	0.00	0.37	**5.31**	1.63
**Reaction 6**	0.25	0.07	0.00	0.00	0.00	0.00	0.11	0.00	0.01	0.00	0.44	**6.25**	2.17

Table S3. Composition of the three model feeds used for mechanistic nanofiltration studies

Solute	pKa	Aliphatic-only mix (mg L^{-1})	Oxalic + Ferulic binary (mg/L)	Full cocktail (mg L^{-1})
**Aliphatic Acids**				
Oxalic Acid	1.27, 4.27	173	600	173
Succinic Acid	4.21, 5.64	141	-	141
Glutaric Acid	4.34, 5.41	67.6	-	67.6
Formic Acid	3.75	591	-	591
**Aromatic Acids/ Aldehydes**				
Gallic Acid	4.40, 8.50, 9.00	-	-	76.85
Benzoic Acid	4.20	-	-	43.6
Syringic Acid	4.55	-	-	39.16
Ferulic Acid	4.58	-	600	114.07
Syringaldehyde	n/a (aldehyde)	-	-	40.26
Vanillin	n/a (aldehyde)	-	-	11.89
Vanillic Acid	4.15	-	-	41.96

Table S4. Mass balance of solid residue and calculated conversion for catalytic depolymerization of poplar lignin (Reactions 1–8).

Reaction Number	Initial Poplar Lignin + Catalyst Weight (mg)	Solid After Depolymerization Reaction (mg)	Conversion (%)
**Initial Poplar**	450	0.00	0.00
**Reaction 1**		170	62.22
**Reaction 2**		253.2	43.73
**Reaction 3**		103.5	77.00
**Reaction 4**		89.5	80.11
**Reaction 5**		247.2	45.07
**Reaction 6**		153.1	65.98

Table S5. GPC calibration data for sodium poly(styrene sulfonate) (PSS) standards

Standard Name	Standard MW (Da)	Log (MW)	UV-RT (min)	Real RT (min)	V_e	k_{av}	log (MW)
**Polystyrene sulfonate sodium salt standards (Agilent)**	246	2.390935	7.767	7.167	7.167	0.269309	2.390935
	1000	3	7.241	6.641	6.641	0.206126	3
	3400	3.531479	7.018	6.418	6.418	0.179339	3.531479
	3970	3.598791	6.861	6.261	6.261	0.16048	3.598791
	4200	3.623249	6.866	6.266	6.266	0.161081	3.623249
	6000	3.778151	6.826	6.226	6.226	0.156276	3.778151
	6520	3.814248	6.696	6.096	6.096	0.140661	3.814248
	10000	4	6.626	6.026	6.026	0.132252	4
	14900	4.173186	6.462	5.862	5.862	0.112553	4.173186
	15000	4.176091	6.558	5.958	5.958	0.124084	4.176091
	20000	4.30103	6.386	5.786	5.786	0.103423	4.30103
	30000	4.477121	6.318	5.718	5.718	0.095255	4.477121

Table S6. Separation factors of rGO membranes for lignin-derived product fractionation.

Membrane Type	S. F. (Aliphatic Monomers) / (HMW)	S.D.	S.F. 30X (Aliphatic Compound) / (HMW)	S.D.	S.F. (Aromatic Monomers) / (HMW)	S.D.	S.F. 30X (Aromatic Products) /(HMW)	S.D.	S.F. (Monomer + Other Low-MW Products) / (HMW)	S.D.	S.F. 30X (Monomer + Other Low-MW Products) / (HMW)	S.D.	S.F. (Oligomers) / (HMW)	S.D.	S.F. 30X (Oligomers) / (HMW)	S.D.
rGO/ PVDF	3.2	1.5	5.8	0.9	2.4	1.1	7.7	2.7	6.2	2.0	2.7	2.1	4.1	2.2	1.4	1.3
rGO/ PES	2.7	0.2	10.8	5.2	2.5	1.1	12.5	7.1	6.4	2.1	4.3	2.1	2.4	1.3	4.6	1.5

Table S7. Permeation measurements with bare PVDF and PES substrates.

Support Type	Nominal support pore size	LMW rejection (%)	HMW (>1.6 kDa) rejection (%)
Bare PVDF	0.2 µm	~0 / below measurable rejection	~0
Bare PES	0.2 µm	~0 / below measurable rejection	~0

Supporting Figures

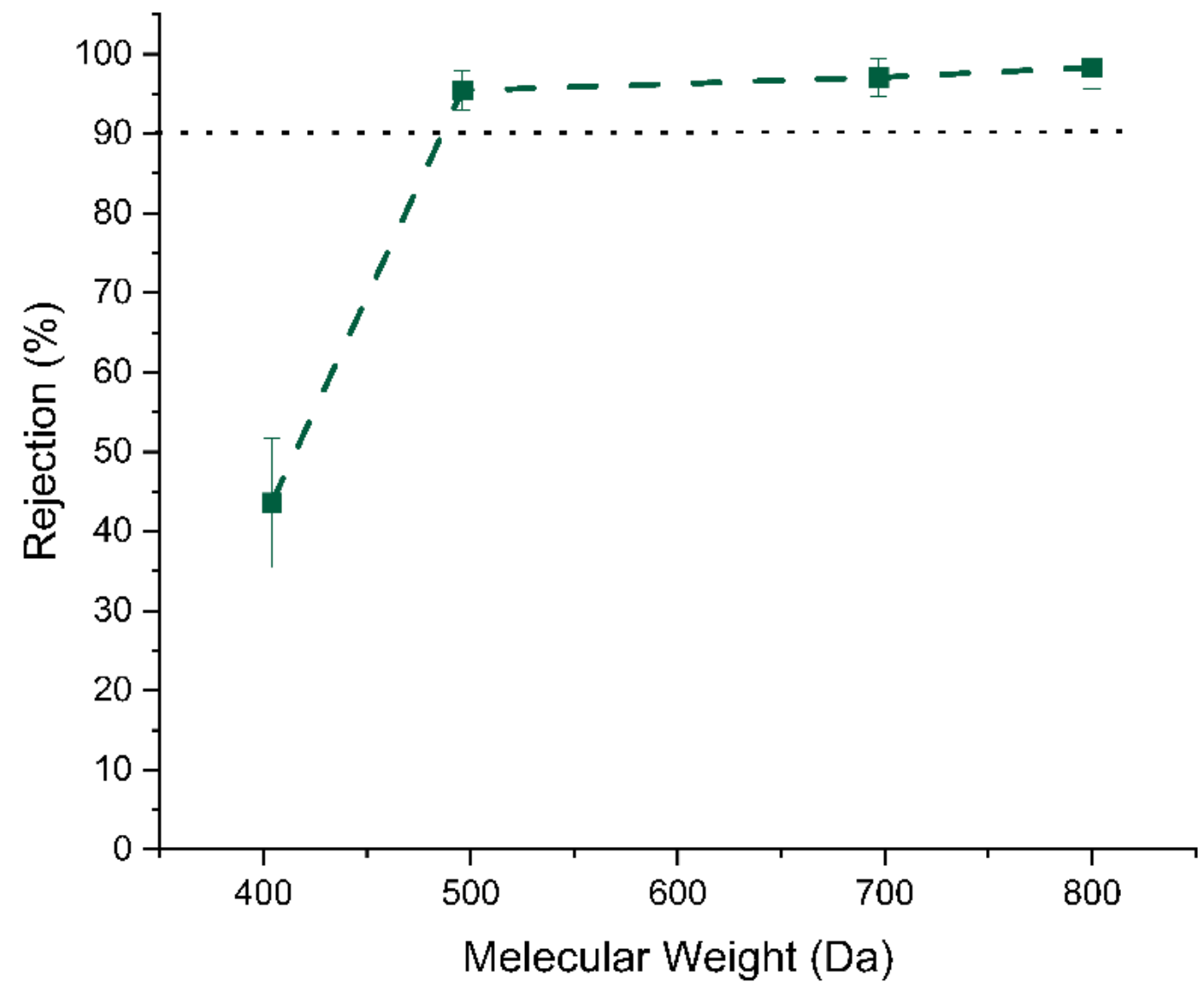

Figure S1. Determination of rGO/MWCO membrane molecular-weight cut-off (MWCO).

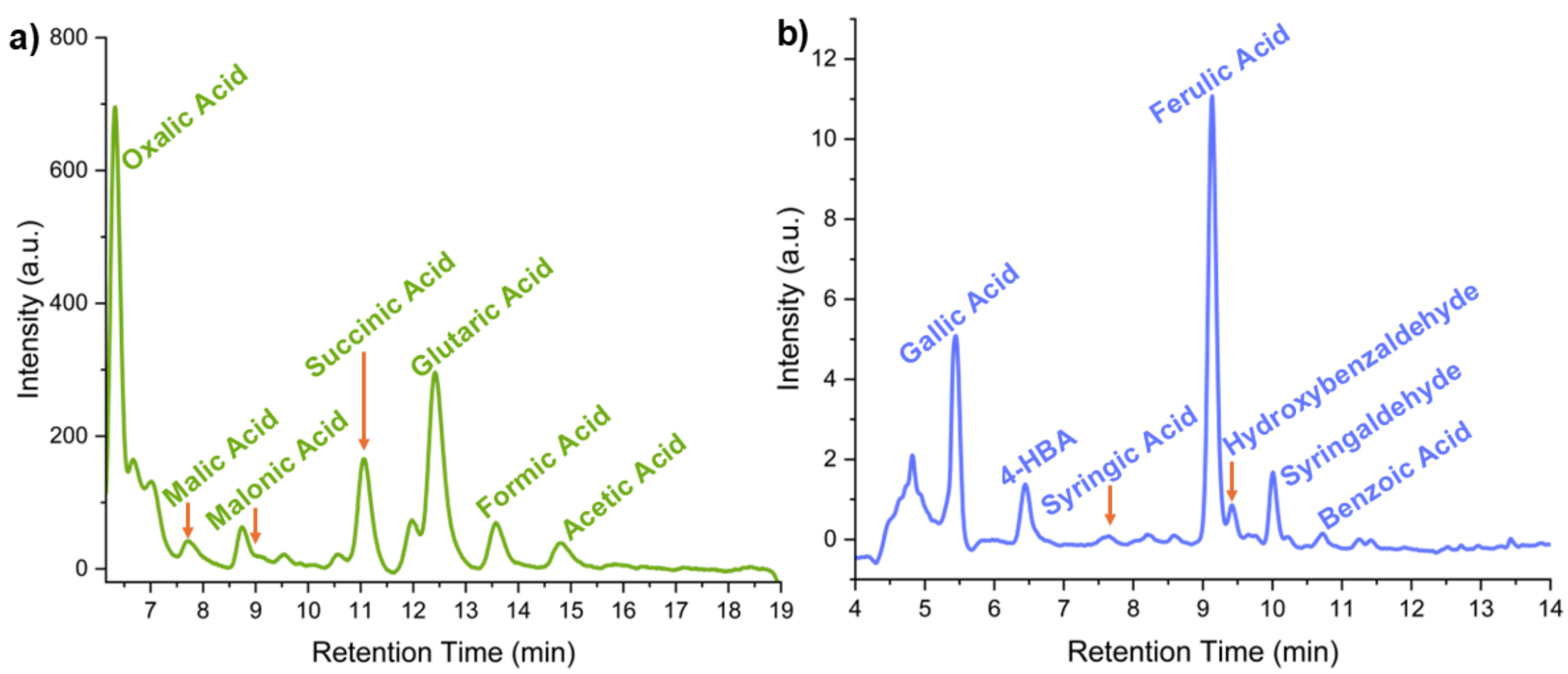

Figure S2. HPLC chromatograms of the Reaction 2 depolymerization liquor (a) Aminex HPX-87H trace highlighting the eight principal aliphatic acids (b) Phenyl-Hexyl trace identifying the ten dominant aromatic monomers.

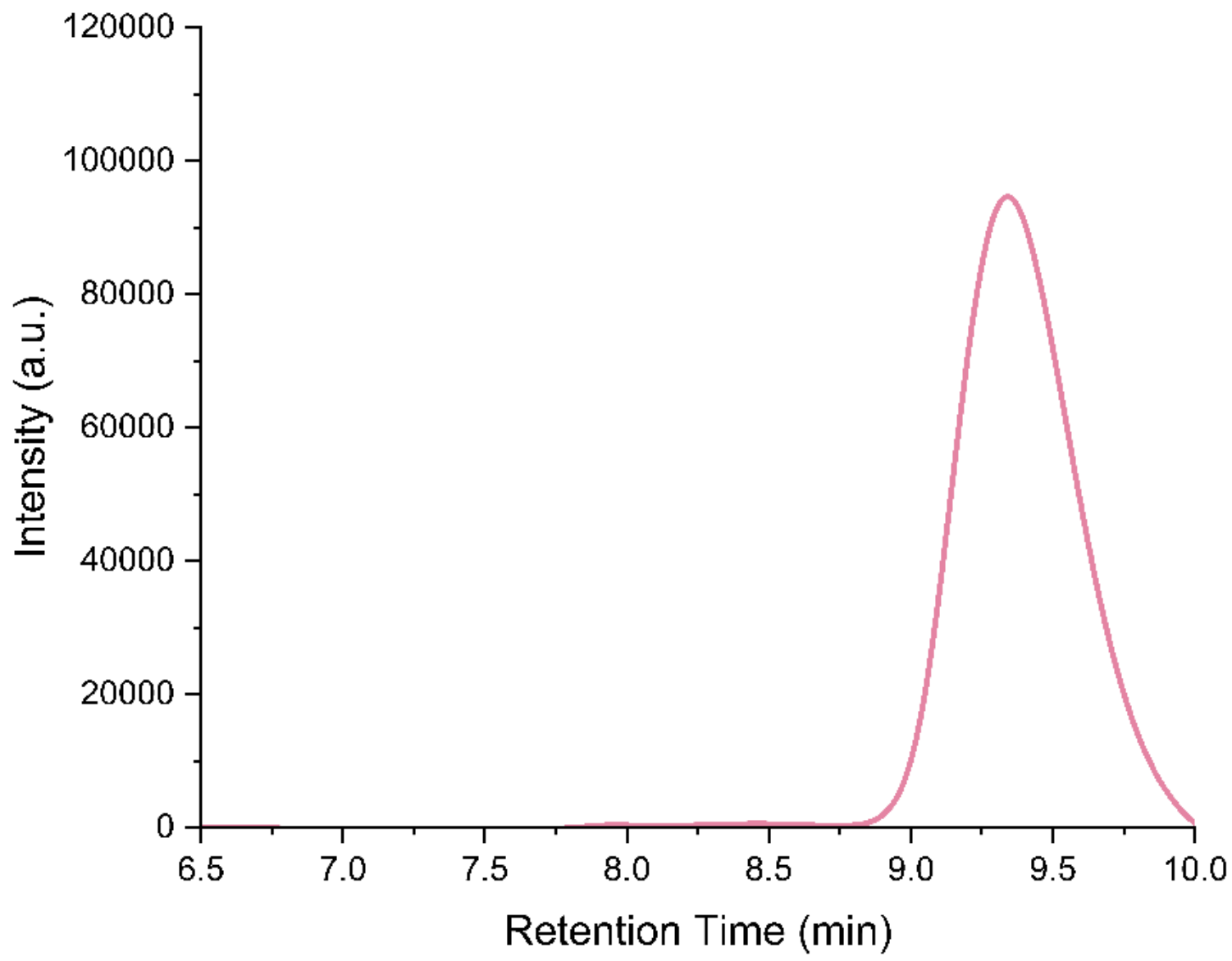

Figure S3. GPC chromatogram of the aromatic acid standard mixture (Table S1) at equal molar concentrations to verify that these monomeric species elute within Zone I window assignement used throughout this work

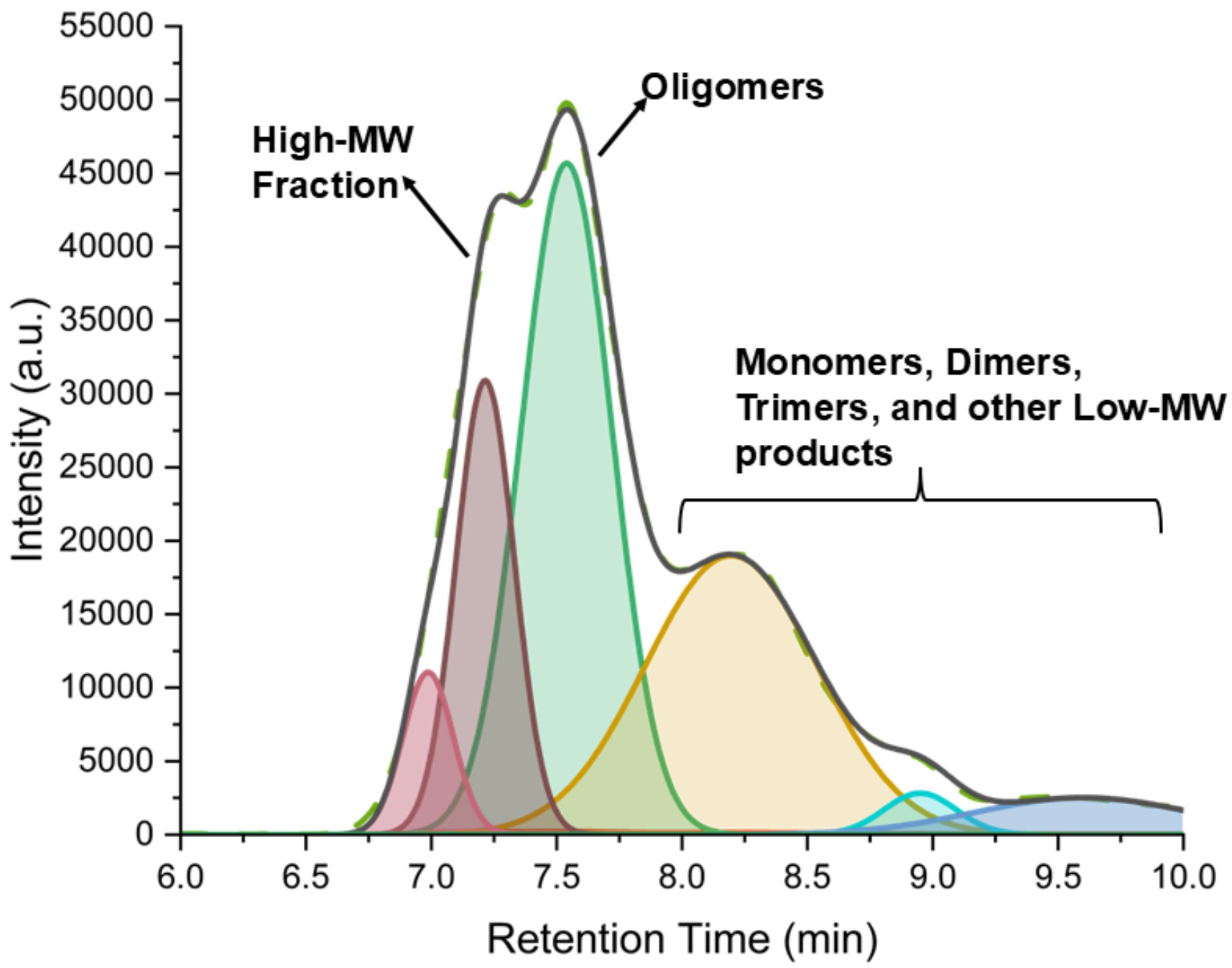

Figure S4. Gaussian deconvolution of a representative GPC trace used to partition the lignin depolymerization product into monomers, dimers/trimers, oligomers, higher oligomers, and high-MW residue; shaded areas are integrated to obtain the relative area for mass/concentration of each group ($R^2 = 99.9\%$)

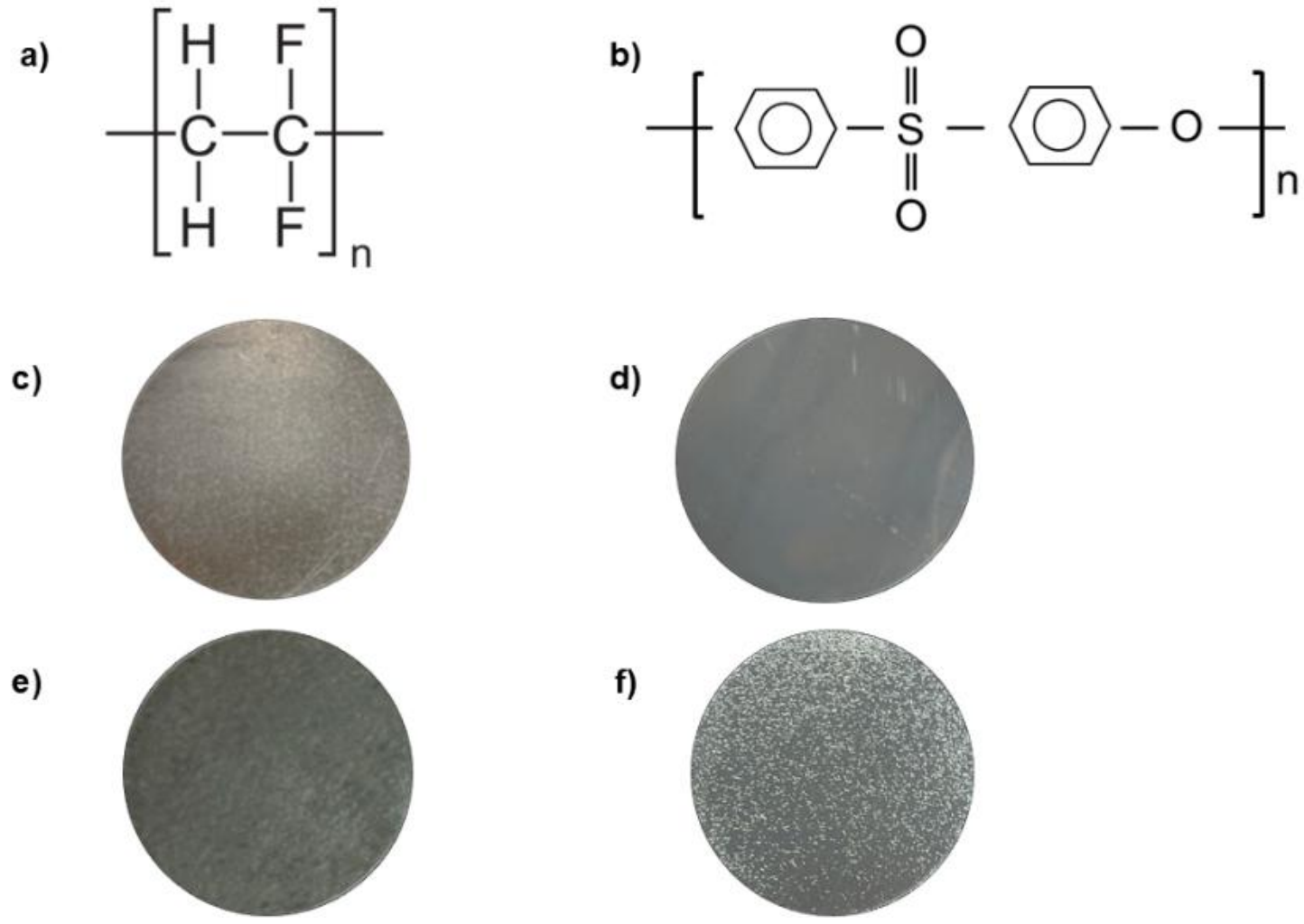

Figure S5. (a) Poly(vinylidene fluoride) (PVDF), (b) poly(ether sulfone) (PES) repeat unit structure of membrane substrates; Photographs of 47 mm in diameter coupons: pristine rGO on PVDF (c) and PES (d) before compaction; and the same rGO|PVDF (e) and rGO|PES (f) after sequential low- and high-pH nanofiltration and long-term nanofiltration (250 h total). No macroscopic defects or fouling deposits are physically detected.

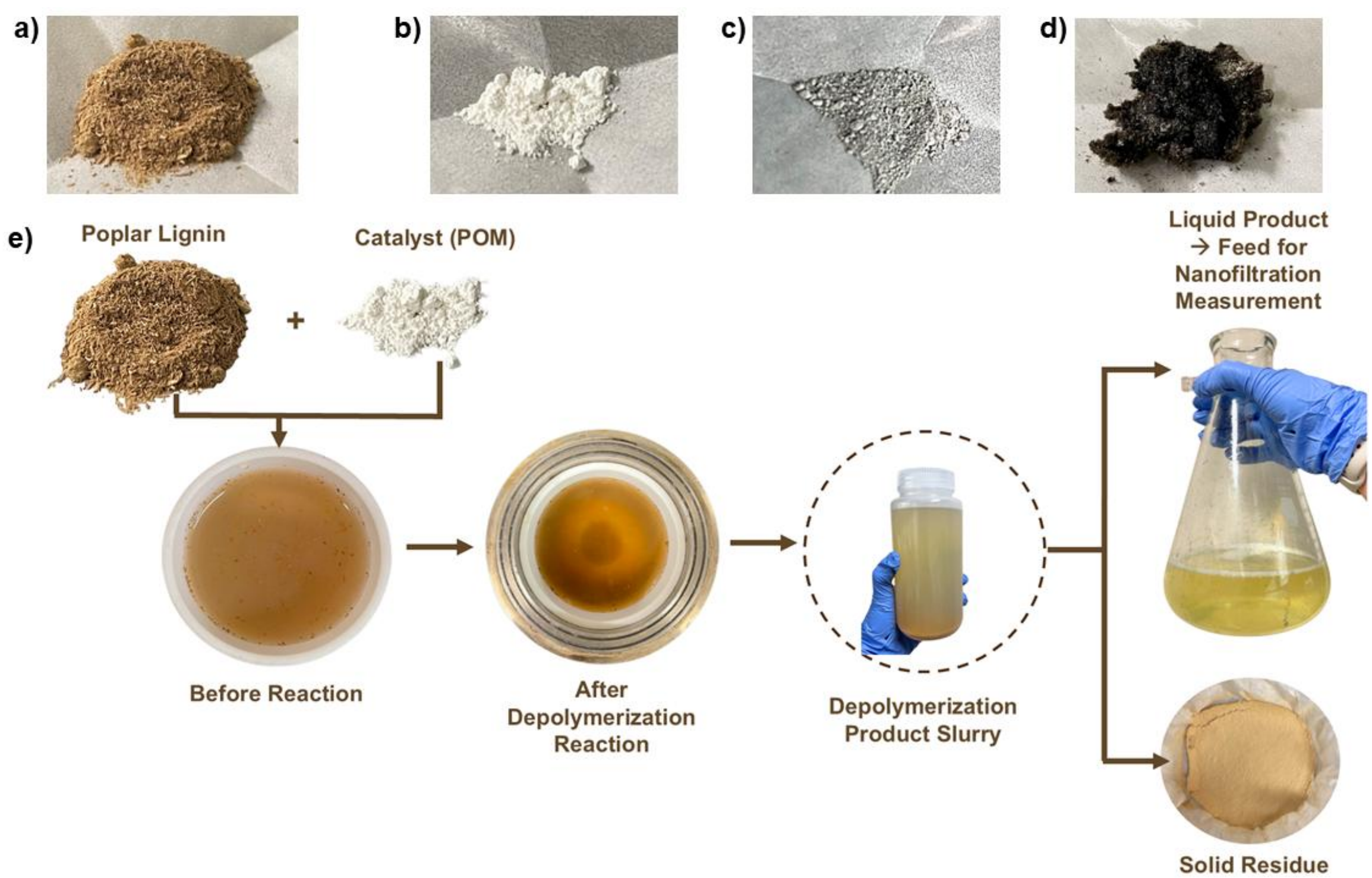

Figure S6. Photographs of reactants, catalysts, and sampling workflow. (a) Poplar lignin powder (feedstock); catalysts used: (b) polyoxometalate (POM), (c) $RuMn_2$, and (d) graphene-oxide (GO) carbocatalyst. (e) Schematic/photo sequence for a representative depolymerization run (Reaction 2): for depolymerization liquor that serves as the nanofiltration feed.

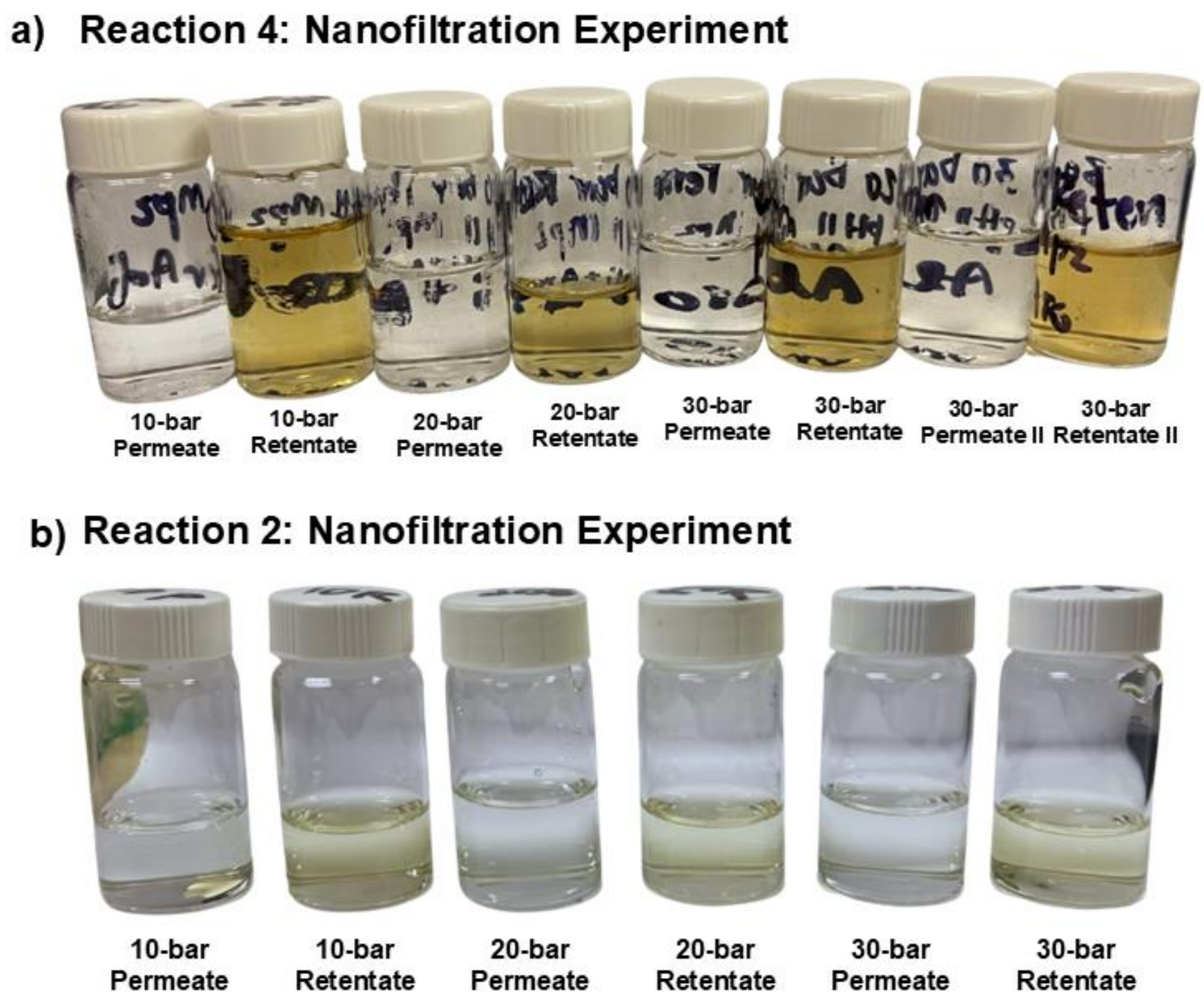

Figure S7. Visual comparison of permeates and retentates obtained by rGO/PVDF nanofiltration at different trans-membrane pressures (TMP = 10, 20, 30 bar) for two representative feeds.

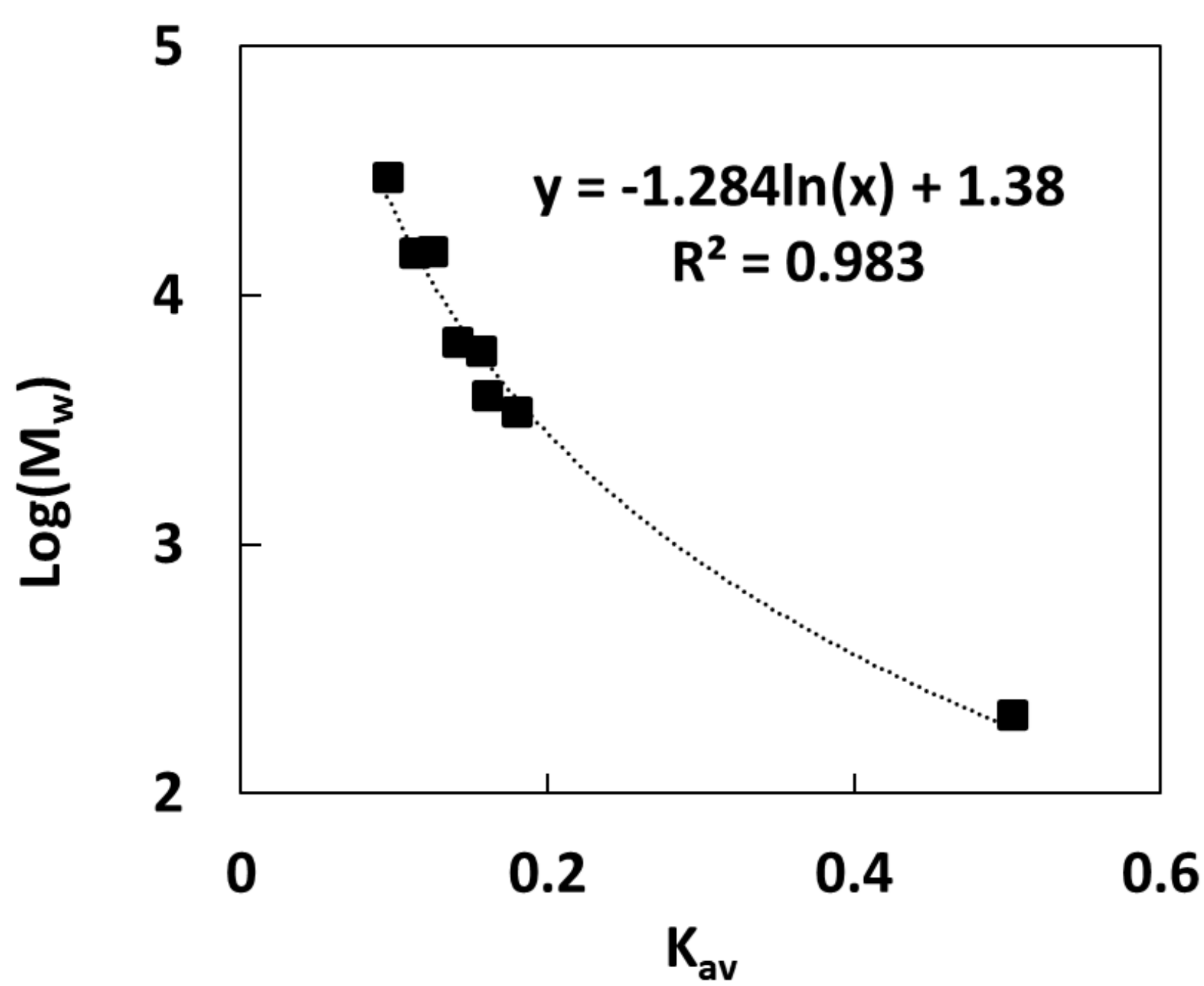

Figure S8. Calibration curve of PLAqua OH columns using poly(styrene sulfonate) (PSS) sodium salt standards of 246, 1,000, 2,000, 3,400, 4,200, 6,000, 10,000, 15,000, 20,000 and 30,000 Da.

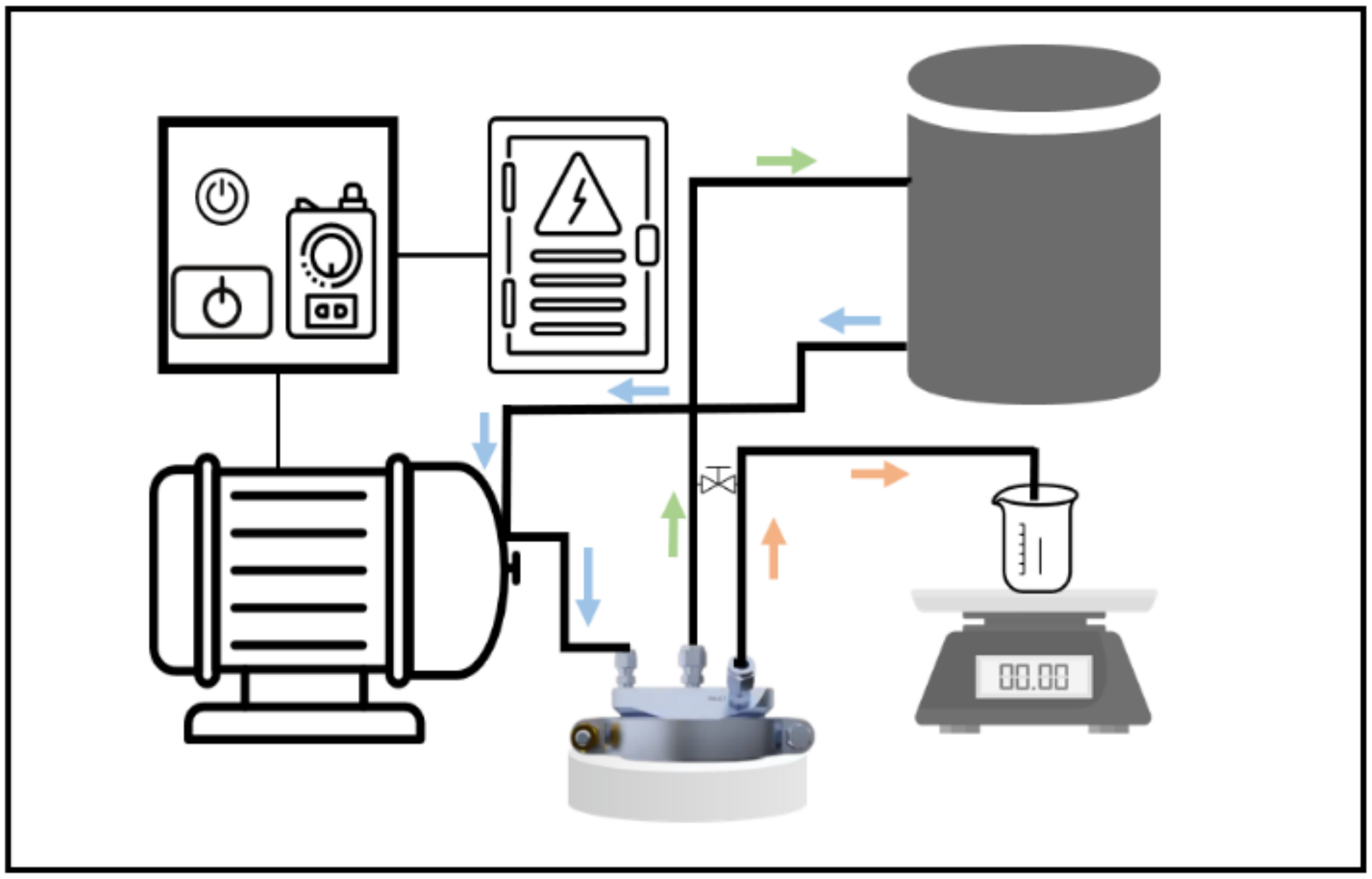

Figure S9. Schematic photograph of the crossflow filtration setup used for membrane permeation measurements. The system consists of a power control box, variable frequency drive (VFD), and feed tank (top row), and a 3-phase motor connected to a 0.5 gal/min Catpump, CF047 membrane cell, and Ohaus 1200 g balance.

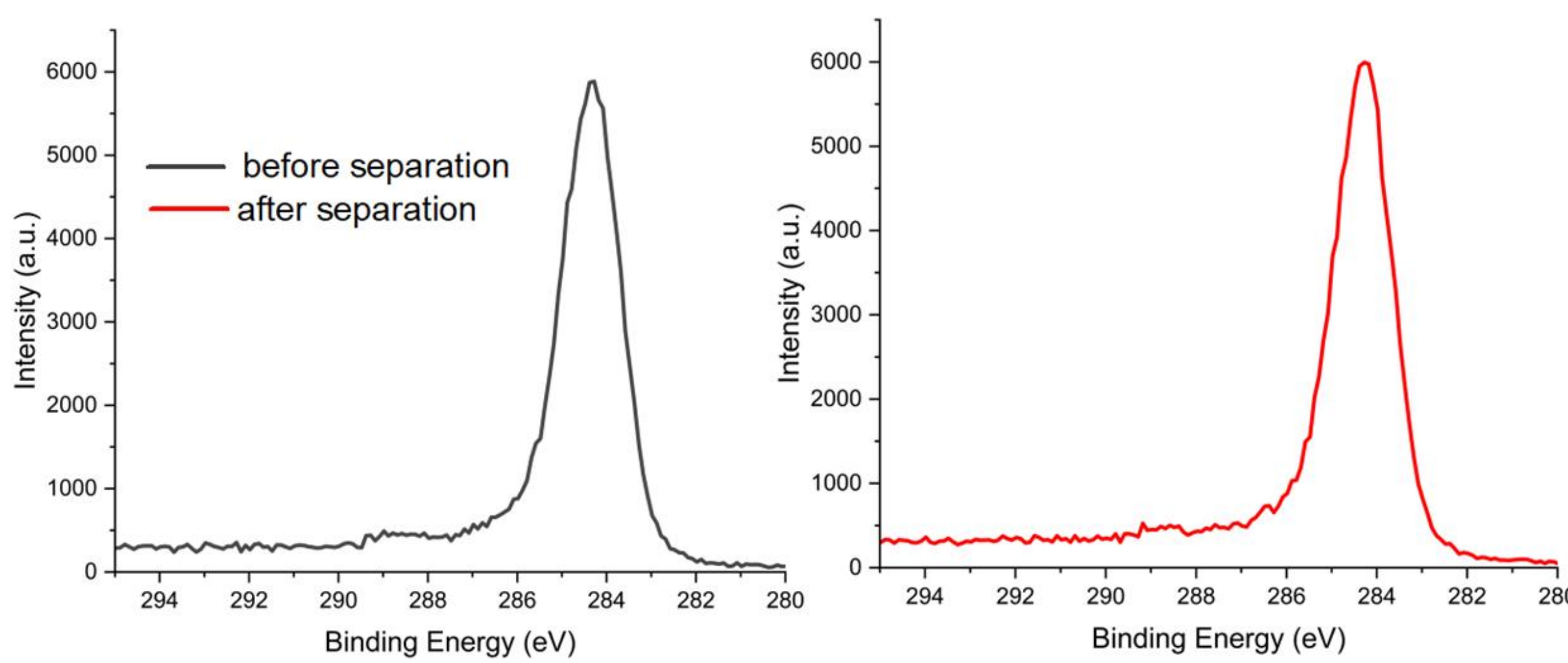

Figure S10. High-resolution C1s XPS spectra of rGO membrane before and after long-term separation.

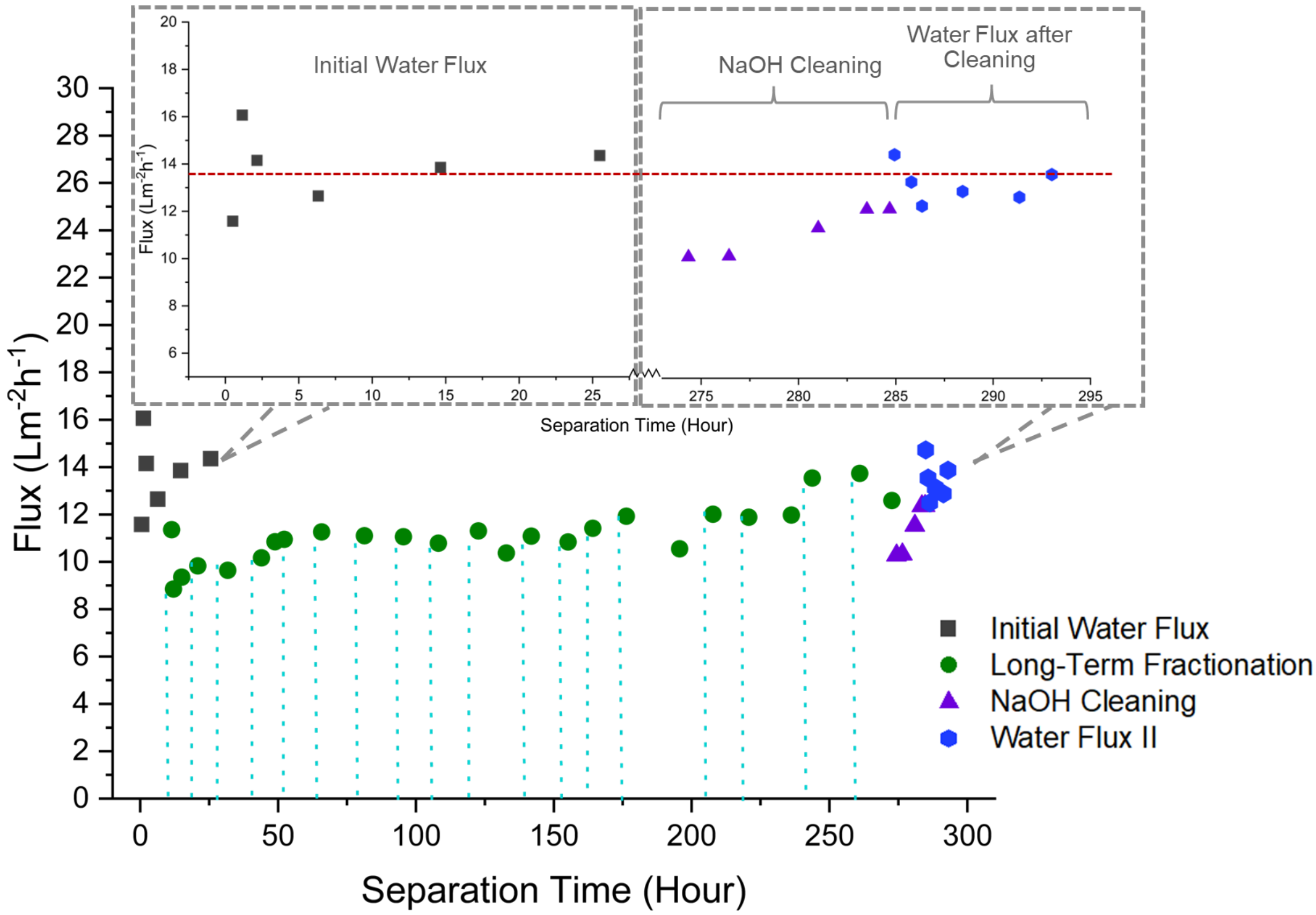

Figure S11. Flux evolution during the longer-term diafiltration run, depicting (1) initial pure-water permeation (black symbols), (2) long-term fractionation (green), (3) NaOH cleaning (purple), and (4) post-cleaning pure-water permeation (blue). Insets provide greater detail of (1), (3) and (4). Blue vertical dashed lines indicate the time points at which DI water was added to the feed reservoir at a volume equal to the permeate withdrawn in the cycle, thus maintaining a constant starting feed volume for the next cycle.
